# Serial monitoring of genomic alterations in circulating tumor cells of ER‐positive/HER2‐negative advanced breast cancer: feasibility of precision oncology biomarker detection

**DOI:** 10.1002/1878-0261.13150

**Published:** 2021-12-20

**Authors:** Andi K. Cani, Emily M. Dolce, Elizabeth P. Darga, Kevin Hu, Chia‐Jen Liu, Jackie Pierce, Kieran Bradbury, Elaine Kilgour, Kimberly Aung, Gaia Schiavon, Danielle Carroll, T. Hedley Carr, Teresa Klinowska, Justin Lindemann, Gayle Marshall, Vicky Rowlands, Elizabeth A. Harrington, J. Carl Barrett, Nitharsan Sathiyayogan, Christopher Morrow, Valeria Sero, Anne C. Armstrong, Richard Baird, Erika Hamilton, Seock‐Ah Im, Komal Jhaveri, Manish R. Patel, Caroline Dive, Scott A. Tomlins, Aaron M. Udager, Daniel F. Hayes, Costanza Paoletti

**Affiliations:** ^1^ Division of Hematology and Oncology Department of Internal Medicine University of Michigan Medical School Ann Arbor MI USA; ^2^ Rogel Cancer Center Michigan Medicine University of Michigan Ann Arbor MI USA; ^3^ Department of Pathology Michigan Medicine University of Michigan Ann Arbor MI USA; ^4^ Michigan Center for Translational Pathology Michigan Medicine University of Michigan Ann Arbor MI USA; ^5^ Department of Computational Medicine and Bioinformatics University of Michigan Ann Arbor MI USA; ^6^ Cancer Research UK Manchester Institute Cancer Biomarker Centre University of Manchester UK; ^7^ Research and Early Development Oncology R&D AstraZeneca Cambridge UK; ^8^ Late Development Oncology R&D AstraZeneca Cambridge UK; ^9^ Research and Early Development Oncology R&D AstraZeneca Waltham MA USA; ^10^ Menarini Silicon Biosystems, Inc. San Diego CA USA; ^11^ Department of Medical Oncology The Christie NHS Foundation Trust and the Faculty of Biology, Medicine and Health The University of Manchester UK; ^12^ Breast Cancer and Early Phase Clinical Trials Teams Cancer Research UK Cambridge Centre UK; ^13^ Sarah Cannon Research Institute/Tennessee Oncology PLLC Nashville TN USA; ^14^ Department of Internal Medicine Seoul National University Hospital Cancer Research Institute Seoul National University College of Medicine Seoul Korea; ^15^ Memorial Sloan Kettering Cancer Center New York NY USA; ^16^ Sarah Cannon Research Institute/Florida Cancer Specialists Sarasota FL USA

**Keywords:** circulating tumor cells, circulating tumor DNA, liquid biopsy, precision medicine, tumor evolution, tumor heterogeneity

## Abstract

Nearly all estrogen receptor (ER)‐positive (POS) metastatic breast cancers become refractory to endocrine (ET) and other therapies, leading to lethal disease presumably due to evolving genomic alterations. Timely monitoring of the molecular events associated with response/progression by serial tissue biopsies is logistically difficult. Use of liquid biopsies, including circulating tumor cells (CTC) and circulating tumor DNA (ctDNA), might provide highly informative, yet easily obtainable, evidence for better precision oncology care. Although ctDNA profiling has been well investigated, the CTC precision oncology genomic landscape and the advantages it may offer over ctDNA in ER‐POS breast cancer remain largely unexplored. Whole‐blood (WB) specimens were collected at serial time points from patients with advanced ER‐POS/HER2‐negative (NEG) advanced breast cancer in a phase I trial of AZD9496, an oral selective ER degrader (SERD) ET. Individual CTC were isolated from WB using tandem CellSearch^®^/DEPArray™ technologies and genomically profiled by targeted single‐cell DNA next‐generation sequencing (scNGS). High‐quality CTC (*n* = 123) from 12 patients profiled by scNGS showed 100% concordance with ctDNA detection of driver estrogen receptor α (*ESR1*) mutations. We developed a novel CTC‐based framework for precision medicine actionability reporting (MI‐CTCseq) that incorporates novel features, such as clonal predominance and zygosity of targetable alterations, both unambiguously identifiable in CTC compared to ctDNA. Thus, we nominated opportunities for targeted therapies in 73% of patients, directed at alterations in phosphatidylinositol‐4,5‐bisphosphate 3‐kinase catalytic subunit alpha (*PIK3CA*), fibroblast growth factor receptor 2 (*FGFR2*), and KIT proto‐oncogene, receptor tyrosine kinase (*KIT*). Intrapatient, inter‐CTC genomic heterogeneity was observed, at times between time points, in subclonal alterations. Our analysis suggests that serial monitoring of the CTC genome is feasible and should enable real‐time tracking of tumor evolution during progression, permitting more combination precision medicine interventions.

## Graphical abstract

Serial noninvasive monitoring of the evolving tumor genome in hormone receptor‐positive metastatic breast cancer on endocrine therapy by liquid biopsy may inform on predictive biomarkers and associated treatments. We show that single‐cell genomic profiling of circulating tumor cells is well suited to complement ctDNA and tissue in identifying mutations, copy number alterations, alteration clonal dominance, and zygosity, enabling real‐time precision oncology.

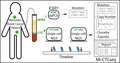

**Abbreviations**
AI: aromatase inhibitor
ATM: ataxia telangiectasia mutated gene
BC: breast cancer
BID: twice daily
BRCA2: BRCA2 DNA repair associated
CNA: copy number alteration
CCND1: cyclin D1
CD45: cluster of differentiation 45
CDH1: E‐cadherin
CK: cytokeratin
CTC: circulating tumor cells
ctDNA: circulating tumor DNA
CxDy: Cycle x, Day y
DAPI: 4′,6‐diamidino‐2‐phenylindole
EpCAM: epithelial cell adhesion molecule
ER: estrogen receptor
ESR1: estrogen receptor alpha gene
ET: endocrine therapy
FDA: Food and Drug Administration
FGFR2: fibroblast growth factor receptor 2
GIST: gastrointestinal stromal tumor
HER2: human epidermal growth factor receptor 2
IDH1: isocitrate dehydrogenase (NADP(+)) 1
IQ: interquartile
IRB: Institutional Review Board
KIT: KIT proto‐oncogene, receptor tyrosine kinase
LBD: ligand‐binding domain
LOH: loss of heterozygosity
MAPK: mitogen‐activated protein kinase
MATCH: Molecular Analysis for Therapy Choice
MBC: metastatic breast cancer
MSI: microsatellite instability
MYC: MYC proto‐oncogene
NCI: National Cancer Institute
NEG: negative
NRAS: NRAS proto‐oncogene
PALB2: partner and localizer of BRCA2
PIK3CA: phosphatidylinositol‐4,5‐bisphosphate 3‐kinase catalytic subunit alpha
POS: positive
scNGS: single‐cell next‐generation sequencing
SERD: selective estrogen receptor degrader
SF3B1: splicing factor 3B subunit 1
SMO: smoothened
TMB: tumor mutation burden
TP53: tumor protein 53
TSC1: TSC complex subunit 1
WB: whole blood
WGA: whole‐genome amplification

## 1 Introduction

Current management approaches for estrogen receptor‐positive (ER POS), HER2‐negative (HER2 NEG) metastatic breast cancer (MBC) commonly consist of endocrine therapy (ET), including the selective ER degrader (SERD), fulvestrant [1]. Fulvestrant’s activity is dose dependent [2, 3, 4] and may be retained even after the acquisition of ligand‐binding domain mutations in *ESR1*, the gene that encodes for ER, which induce ligand‐independent signaling [5]. However, pharmacologically, fulvestrant requires large volume intramuscular injection, so dose escalation in its current formulation is difficult. Oral SERDs have entered clinical trials [6] and patients from a phase I trial of one such oral SERD candidate, AZD9496, are the focus of this study. The candidate drug only showed limited activity and all patients reported here had progressive disease as their best response. However, in‐depth study of these patients’ CTCs can shed light on CTC genomic actionable biomarkers, intrapatient heterogeneity, and clonal mechanisms of progression in metastatic ER POS, HER2 NEG breast cancer.

Ideally, ET pharmacological agents should be developed with companion predictive and pharmacodynamic tumor biomarkers that can be non‐invasively tracked over the course of treatment. Real‐time detection and monitoring of genomic alterations in tissue are problematic, since relevant archived tumor‐derived material is collected years or months before, at the time of primary or first metastatic diagnosis, and serial tissue biopsies are invasive and logistically difficult [7, 8]. Liquid biopsies, centered around detection of cell‐free circulating tumor DNA (ctDNA) and circulating tumor cells (CTC), have overcome these difficulties by allowing non‐invasive detection/monitoring of genomic alterations [9]. However, the benefits of ctDNA may be dampened by some technical limitations and assay variability due to the often‐low tumor DNA fraction [10, 11]. Further, since ctDNA represents a composite view of bulk tumor DNA in circulation, its ability to discern cancer clonal and subclonal architecture and intrapatient heterogeneity, a recognized cause of therapy resistance [12], is also limited.

Circulating tumor cells are shed by the tumor and are thought to represent the transit phase of the invasion metastasis cascade or, in the context of recurrent metastatic disease, continued spread [13, 14]. CTC enumeration is prognostic in several cancers, such as breast, prostate, colorectal, and lung [15, 16, 17, 18, 19, 20, 21, 22]. In addition to CTC enumeration, their phenotyping and genotyping may provide additional biologic and perhaps clinically relevant information [23, 24]. Yet, clinical application of CTC‐derived precision oncology biomarkers, despite having the potential to provide additional clinically relevant information, remains largely unexplored. We have previously reported CTC enumeration, phenotype, and ctDNA *ESR1* results from our AZD9496 oral SERD phase I trial [25]. Here, we report on the genomic analysis of CTCs at single‐cell resolution by scNGS at two time points and compare it to ctDNA results for *ESR1* ligand‐binding domain mutations (LBD) in patients who participated in this trial.

## 2 Materials and methods

### 2.1 Study design and objectives

This was a correlative study using specimens from the NCT02248090 phase I trial, a multicenter international study investigating safety and tolerability of the oral SERD AZD9496 in ER POS/HER2 NEG metastatic or locally recurrent breast cancer [6]. CTC enumeration, phenotyping, and ctDNA ESR1 mutational analysis have been previously reported [25]. The phase I study as well as collection and profiling of liquid biopsy samples for the successive correlative work, including this analysis, has been conducted in accordance with the principles of the International Conference on Harmonization Guidelines for Good Clinical Practice and the Declaration of Helsinki. The study methodologies were approved by the local ethics committee (Institutional Review Board, IRB). All experiments were undertaken with the understanding and written consent of each subject.

### 2.2 Patient eligibility and AZD9496 dosing

Detailed eligibility criteria, conduct, and results of the main clinical trial, in which participating patients received escalating doses until disease progression or unacceptable toxicity, have been previously described [6]. Briefly, women with ER POS/HER2 NEG MBC having undergone ≥ 6 months of ET were eligible while those having received > 2 lines of chemotherapy were excluded.

### 2.3 CTC collection and processing

Details of blood collection, processing, and CTC enrichment and purification using CellSearch^®^ and DEPArray™ have been previously described [24, 25]. Briefly, blood was collected into three separate CellSave tubes (Menarini Silicon Biosystems S.p.A., Bologna, Italy) within 28 days prior to initiating therapy (screening), at initiation (Cycle 1, Day 1—C1D1), and then either C1D15 or at treatment discontinuation. Blood from the three tubes was pooled, mixed, re‐aliquoted to 7.5 mL, and processed for CTC enrichment as previously described using the CellSearch^®^ CTC enrichment system (Menarini Silicon Biosystems) [25, 26]. CellSearch^®^ cartridges were initially stored at 4 °C in the dark post‐CellSearch^®^ processing. However, data reported during the conduct of the trial suggested that storage in glycerol at −20 °C is superior, and subsequent specimens were stored in that manner [27]. Individual CTCs were obtained by processing CellSearch^®^ cartridge contents on the DEPArray™ system (Menarini Silicon Biosystems) per the manufacturer's instructions, at either Menarini Silicon Biosystems’ central laboratory or at the University of Michigan, as previously described [24]. Individual selected cells passing pre‐specified criteria (DAPI and cytokeratin positivity, CD45 negativity) were routed for isolation and recovery via dielectrophoretic cell sorting by DEPArray™ [28]. Individual CTC were lysed and DNA was whole‐genome‐amplified (WGA) using the Ampli1 WGA kit (Menarini Silicon Biosystems) with MseI digestion per the manufacturer's instructions. WGA DNA quality control was performed using Ampli1 QC Kit [29], and low‐quality DNA cells (< 3 QC bands) were not pursued for further genomic analysis.

### 2.4 CTC single‐cell genomic analysis

A maximum of 96 individual CTCs per CellSearch^®^ cartridge can be recovered from DEPArray™. Purified CTCs were WGA’d with the goal of obtaining 10 high‐quality CTC from each patient per time point for downstream genomic analysis with additional cells added for select interesting cases (#17, #26, and #34, up to 19 total cells per time point). ScNGS was performed as previously described [24, 30, 31, 32, 33]. Briefly, 20 ng of WGA’d DNA per CTC underwent library construction with a targeted NGS custom AmpliSeq panel (Ion Torrent, Thermo Fisher Scientific, Waltham, MA, USA). The panel targets 138 cancer‐related genes selected based on pan‐solid tumor genomic data analysis that prioritized recurrent and/or targetable cancer alterations. The panel (Table S1) is an enhanced version of the Oncomine Cancer Panel (Thermo Fisher) used in the NCI‐MATCH basket trial [30, 34]. Approximately one‐third of panel amplicons are negatively affected due to WGA MseI digestion. Templating, sequencing, and data analysis were performed on the Ion Torrent Chef, S5 Prime Gene Studio, and Torrent Suite version 5.0.2, respectively.

### 2.5 Data analysis and variant prioritization

Variant and copy number (CN) annotation, filtering, and prioritization were performed essentially as previously reported using validated in‐house pipelines [24, 30]. Candidate somatic variants called by the Torrent Browser and annotated with ANNOVAR were filtered to remove synonymous or non‐coding variants, those with read depths (FDP) < 10, variant read frequencies < 0.10, extreme skewing (> 5‐fold) of forward/reverse read ratio calling the variant, or indels within homopolymer runs > 4 nucleotides long. Called variants were filtered using a panel‐specific, in‐house blacklist. Variants reported at population allele frequencies > 0.5% in EXAC or 1000 Genomes (KG) databases, were considered germ line variants unless occurring at a known cancer hot spot. Somatic variants passing the above filters were then visually confirmed in the Integrated Genome Viewer (IGV, Broad Institute) as were the same regions in samples from the same patient where the variant was not called, in order to confirm both adequate coverage and absence of the variant. Variants located at the last mapped base (or outside) of amplicon target regions, those with the majority of supporting reads harboring additional mismatches, those in repeat‐rich regions (likely mapping artifacts), or those occurring exclusively in one amplicon if overlapping amplicons cover the position, were excluded. We have previously confirmed that these filtering criteria identify variants that pass Sanger sequencing validation with > 95% accuracy [31, 35].

We prioritized as putative driving alterations those that were deleterious in tumor suppressor genes (nonsense, frameshift, deletion), recurrent ‘hotspot’ mutations (in the ‘Curated set of non‐redundant studies’ cohort, cbioportal.org or those with solid literature evidence) or those with OncoKB driver annotation in oncogenes or tumor suppressors or amplifications in oncogenes. Somatic mutations without support from any of those categories were designated as VUS. We define homozygous mutations as having a variant read fraction of 0.10–0.95 and homozygous variants at > 0.95. Copy number analysis from total amplicon read counts provided by the CoverageAnalysis plugin (version v5.0.2.0) was performed essentially as previously described using a validated approach [31, 36] with adaptations for single‐cell sequencing. Specifically, we retained only well‐performing amplicons (with > 100 reads in a set of 10 individual WBC samples) in order to exclude amplicons lost due to their containing the MseI restriction site.

Copy number alterations (CNAs) were calculated as normalized read counts per amplicon divided by the normalized mean read count of the same amplicons from the set of unrelated 10 WBC samples sequenced by scNGS with the same gene panel, yielding a copy number ratio for each amplicon. Gene‐level copy number estimates were determined by taking the coverage‐weighted mean of the amplicon ratios [36]. Genes with a copy number estimate < 0.25 or > 4 were considered to have high‐level loss or gain, respectively. Normal epithelial cells in circulation were defined as CTC (DAPI and cytokeratin POS, CD45 NEG) but harboring no apparent driver truncal or subclonal alterations as detected with our panel. Shared somatic alterations were used to confirm CTC belonging to the same patient‐clone [37].

### 2.6 Nomination of actionable precision medicine alterations

Nomination of potentially actionable alterations with MI‐CTCseq was performed by first categorizing alterations present in > 1 CTC within a patient as biomarkers for treatments belonging to one of four tiers of evidence [30, 38]: (a) FDA‐approved for the current indication (MBC), (b) FDA‐approved drugs recommended for off‐label use by practice guidelines for this or other cancer indications, (c) investigated as targeted therapies in interventional biomarker‐driven clinical trials in any cancer type actively recruiting at the time of reporting, and (d) having preclinical evidence/biological plausibility of actionability. This classification is based on commonly used principles on actionability reporting guidelines. We then developed a novel platform for targetability reporting that refines the classical tier system above via the inclusion of subtiers that factor in the clonality of the cell population harboring the alteration, that is, the fraction of total CTC positive for the alteration (Subtier A: ≥ 80%, B: 50–80%, C: < 50%, of total CTC). Additionally, clinically relevant zygosity status of alterations in tumor suppressors and oncogenes is reported (requiring again that the zygosity status be present in > 1 CTC if heterogeneity of zygosity is present for an alteration within a patient). The best level of evidence (a. highest priority tier, b. highest clonal fraction, and c. homozygosity/complete deletion) was used as the final assignment for patients with more than one actionable alteration.

Fish plot analysis for patient #26 was performed in r (version 4.1.0) as reported by Miller et al. [39] under the assumptions of clonal parentage in the order: (a) CDH1 mutation, (b) ESR1 heterozygous mutation, (c) ESR1 homozygous mutation.

### 2.7 Statistical analysis

Means and standard deviations were used to report CTC quality for WGA and sequencing results. Differences in copy number estimates between individual genes in different cell populations were detected by the Student’s *t* test if normally distributed, otherwise the Mann–Whitney test was used. Panel‐wide copy number clustering for different cell populations was performed with unsupervised hierarchical clustering by sample (Cluster 3.0, correlation uncentered similarity metric, centroid linkage method) and visualized with Java Treeview in log2 scale with the middle value for each gene across samples set at 1 (2 copies, diploid).

## 3 Results

### 3.1 Patient cohort and study design

**Fig. 1:**
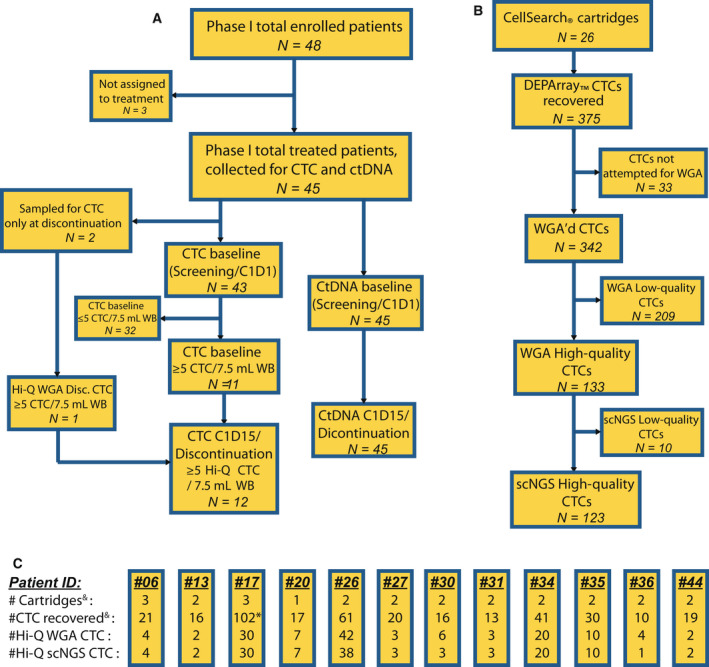
Consort diagram of AZD9496 phase I study patients and their CTC and ctDNA obtained at each time point. (A) Consort diagram of patients attempted for CTC and ctDNA collection showing cases with informative analytes for each time point. (B) Flowchart of the total number of the cartridges and CTCs in each of the categories in panel 1A at each processing step. (C) Details of CTC isolation for each patient at each processing step, showing numbers of CellSearch^®^ cartridges assayed, DEPArray™‐recovered cells, high‐quality whole‐genome amplified cells, and high‐quality scNGS cells. ^&^ One cartridge (nine recovered cells) from panel 1B, not included here belongs to a discontinuation‐only patient with no high‐quality CTC who was excluded from further study. * 33 of 102 CTCs for patient #17 were not WGA’d, to minimize unnecessary costs. CTC, circulating tumor cells; ctDNA, circulating tumor DNA; CxDy, Cycle x, Day y; Hi‐Q, high quality; scNGS, single‐cell next‐generation sequencing; WB, whole blood; WGA, whole‐genome amplification.

Specimens were collected from advanced breast cancer patients pre‐treated with ET, including aromatase inhibition (AI). These patients were enrolled in the phase I AZD9496 trial (NCT02248090, Fig. 1A). We have previously reported clinical trial outcomes and quantification of circulating biomarkers (CTC enumeration and phenotyping as well as ctDNA mutant *ESR1* detection) [6, 25]. Of the 48 enrolled patients, three were not eligible, two did not have blood drawn at baseline, and 11 of the remaining 43 (26%) had ≥ 5 CTC/7.5 mL whole blood (WB) (Fig. 1A) [6]. These 11 patients, as well as the two other patients who did not have baseline specimens drawn, had specimens collected at or near the time of treatment discontinuation. Twelve of these 13 patients had ≥ 5 high‐quality CTC/7.5 mL WB. In‐depth genomic analysis of individual CTC from these 12 patients is the focus of this report.

### 3.2 CTC single‐cell targeted next‐generation sequencing

The number of available cartridges, recovered CTC, high‐quality CTC, and CTC that were ultimately successfully sequenced is provided in Fig. 1B. We attempted isolation of individual CTC using the DEPArray™ system on 26 archived CellSearch^®^ cartridges from 11 patients with available CTC at both time points and two patients at discontinuation only. We recovered 375 single CTC, 342 of which underwent whole‐genome amplification (WGA) with 133 of those (39%) yielding high‐quality WGA product. These individual CTCs underwent comprehensive, multiplexed, amplicon‐based, targeted DNA scNGS, which was successful in 123 (92.5%) of them (Fig. 1B,C). Ultimately, we sequenced high‐quality CTC from 12 patients, 11 of whom from both baseline (screening or cycle 1, day 1 (C1D1)), and later time point (C1D15 or discontinuation of therapy). One additional sequenced patient did not have a baseline blood draw but did have a discontinuation sample that contained ≥ 5 CTC/7.5 mL WB of high WGA quality.

At the initial enrollment period, CTC were stored in the CellSearch^®^ cartridges at 4 °C without additives, but during enrollment, storage of CTC in glycerol at −20 °C was described [27]. Therefore, overall, CTC from eight and four of the 12 eligible patients were stored in CellSearch^®^ cartridges at 4 °C or glycerol at −20 °C, respectively. A comparison of these two methods, described in detail in Supplementary Materials (Fig. S1, Table S2), suggested that storage in glycerol at 20 °C may be preferable for long‐term storage, but of little added benefit in the short term.

**Fig. 2:**
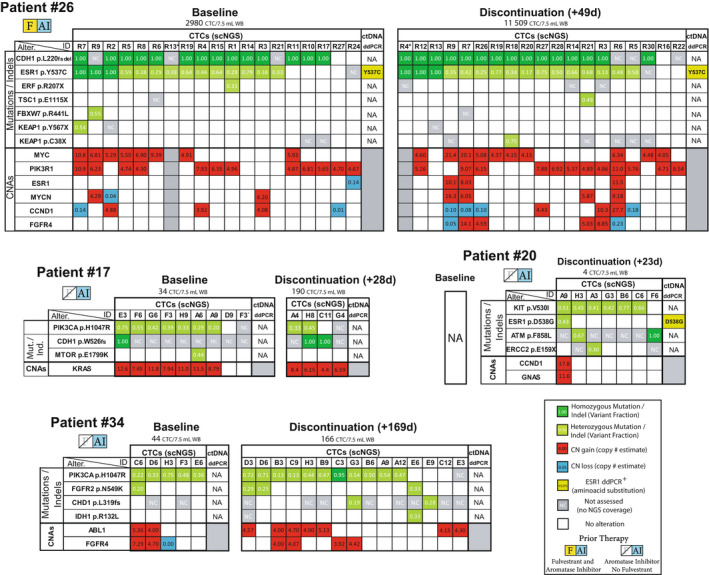
Integrative heatmap of putative driver genomic alterations detected by CTC scNGS and ctDNA ddPCR. Comprehensive genomic analysis of individual CTCs in four of the 12 patients. For each patient, total CTC count at each time point is shown. Columns represent individual CTCs. White boxes indicate adequate coverage and absence of the variant. For mutations/indels (top of each table), colored boxes indicate mutation presence, with dark and light green representing homo‐ and heterozygous mutations, respectively. Numbers inside colored boxes represent the variant read fraction. Gray ‘NC’ boxes indicate no NGS coverage for that position. Mutations private to single cells are shown only for select cases. For CNAs (bottom of each table), estimated copy number is calculated back from the log2(tumor/normal copy ratio) value. High‐confidence, high‐level copy changes (< 0.25 or > 4.0 estimated copies) are shown, with red and blue representing amplifications and deletions, respectively. Only high‐level CNAs present in > 1 CTC are shown. * Cells with suboptimal CNA data (Patient #26, Baseline cell R13, Discontinuation cell R4). Patient #17 baseline CTC F3’ is from C1D1 whereas the rest from screening. DdPCR *ESR1* LBD mutation presence/absence and amino acid change are shown at the right end of each table. Later time points for ddPCR consist of C1D15 samples only. NA, not available (Patient #20 was only drawn for CTC at discontinuation). Empty gray box indicates low‐quality copy number data. Not all CTC are shown for all patients. AI, aromatase inhibitor; Alter., genomic alteration; CNA, copy number alteration; CTC, circulating tumor cells; ctDNA, circulating tumor DNA; CxDy, Cycle x, Day y; ddPCR, digital droplet polymerase chain reaction; F, fulvestrant; Ind., indel, insertion/deletion; LBD, ligand‐binding domain; Mut., mutation; NA, not available; NC, no coverage; scNGS, single‐cell next‐generation sequencing; WB, whole blood.

We sequenced the 123 high‐quality CTCs from the 12 evaluable patients (mean 10.3, range 1–38 CTC per patient) to a mean depth of 983× (IQ range 837–1126×, Table S3) and identified a total of 67 high‐confidence, somatic, putative cancer driver mutations, short insertions/deletions (indels) and high‐level copy number alterations (CNAs). Of the 12 patients, the four most illustrative cases are shown in Fig. 2 and the rest in Fig. S2. These genomic alterations were distributed at a median of five per patient (range 0–17). Only 30 of the 67 alterations (45%) were found in multiple cells within a patient (i.e., truncal or subclonal alterations) at a median of three such truncal/subclonal alterations per patient (range 0–8). The rest were harbored privately by individual CTC.

Strategies to enrich CTC from whole blood that are based on epithelial cell surface markers, like the one we used (CellSearch^®^: EpCAM and Cytokeratins 8, 18 and 19), have been reported by our group and others to also capture a minority of epithelial cells that harbor no apparent cancer‐driving genomic alterations, and which could in fact be normal epithelial cells [24]. In line with these observations, we identified 9/123 cells (7.3%) of apparent epithelial origin (median 1, range 0–2 per patient) from six patients in this cohort that harbored no driver mutations/indels or high‐level CNAs assayed by our comprehensive gene panel (Fig. 2 and Fig. S2).

### 3.3 ESR1 mutation concordance between CTC and ctDNA

**Fig. 3:**
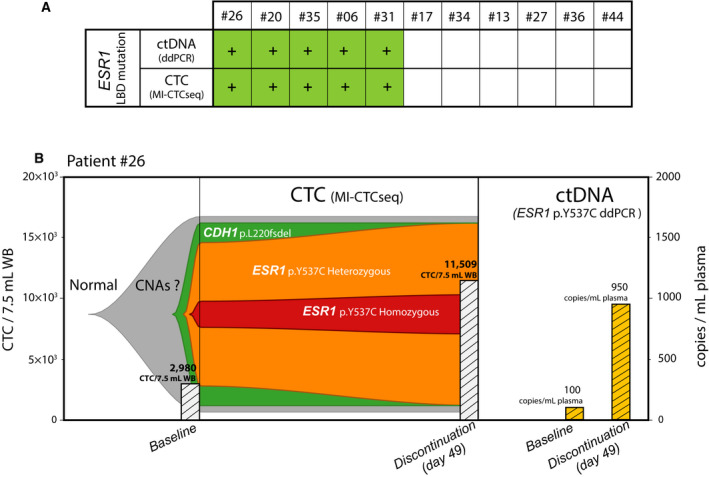
CTC scNGS recapitulates ctDNA findings and elucidates the tumor subclonal evolution. (A) Concordance for the presence of identical *ESR1* LBD mutation between CTC and ctDNA for each of the 11 evaluable patients is shown. Green (+) represents mutation presence in at least one time point. (B) Comparison of CTC scNGS and ctDNA ddPCR analysis for patient #26. Left, fish plot model of tumor clonal heterogeneity and evolution between baseline at the start of trial and at trial discontinuation at day +49. This patient did not have a confirmed response to AZ9496. The most parsimonious clonal parentage assumptions are made for the *CDH1* mutation as an early, nearly truncal event, followed by the arisal of a uniallelic, heterozygous *ESR1* hotspot mutation from AI therapy, later undergoing a loss‐of‐heterozygosity event to become homozygous. CTC enumeration in cells/7.5 mL whole blood is plotted for each time point in the bar graph. Right, information obtained from bulk ctDNA ESR1 LBD mutation ddPCR is limited to changes in mutant DNA concentration in copies/mL plasma, plotted in the bar graph. CTC, circulating tumor cells; ctDNA, circulating tumor DNA; ddPCR, digital droplet polymerase chain reaction; LBD, ligand‐binding domain; scNGS, single‐cell next‐generation sequencing; WB, whole blood.

*ESR1* ligand‐binding domain (LBD) mutations, commonly arising during clinical acquired resistance to aromatase inhibitors (AI), confer ligand‐independent ER activity [5]. Importantly, *ESR1* LBD mutant tumors retain partial response to fulvestrant, which underscores the clinical importance of their detection and monitoring by liquid biopsy [5, 40]. In our prior publication, *ESR1* LBD mutations detected in ctDNA were reported in the larger set of patients in this phase I trial [25]. Six of the 12 (50%) patients in the current study harbored these ctDNA mutations (Fig. 2 and Fig. S2). (CTC scNGS data from one patient were discarded due to strong indications from her CTC genomic alterations that the cells belonged to a different patient, which highlights the exquisite ability of NGS methods to eradicate sample mislabeling issues.) In the 11 remaining evaluable patients, there was 100% concordance between CTC and ctDNA *ESR1* LBD mutation detection (Fig. 3A). Thus, 5/11 patients positive for *ESR1* mutations in ctDNA had CTCs harboring the same mutation, whereas the six patients without *ESR1* ctDNA mutations were *ESR1* mutation‐negative in their CTCs as well. *ESR1* mutations were generally mutually exclusive with MAPK pathway mutations supporting the two as different ET resistance mechanisms, as has been reported [41]. One of the ctDNA *ESR1* mutation‐NEG patients (#36), previously reported to harbor a p.Y537C mutation in her tumor tissue [25], was mutation‐NEG in her CTC as well, in agreement with ctDNA. Taken together, these data support CTCs as sources of genomic cancer biomarkers that may have at least equal validity to ctDNA‐derived markers. Our observations also raise interesting questions on the contribution of CTCs vs. the bulk tumor mass as cells of origin for ctDNA.

### 3.4 Dynamic changes in ESR1 mutation levels in CTC and ctDNA over time

We previously reported that 5 of the 6 baseline *ESR1* mutation‐POS patients had dynamic changes (of > 50%) in ctDNA *ESR1*‐mutant fraction (of total plasma cell‐free DNA) between baseline and C1D15 of AZD9496 treatment [25]. Only one of these five patients, #26, had enough CTCs in both the baseline and discontinuation samples to enable a comparison between ctDNA and CTC mutant *ESR1* levels and their fluctuations over time. This patient, #26, had lobular BC that harbored a somatic *CDH1* p.L220 frameshift deletion (fsdel) and had been previously treated with fulvestrant and AIs. The patient had an elevated baseline CTC count of ~ 3000 CTC/7.5 mL WB and quickly progressed on the highest study dose (AZD9496 600 mg BID) to a remarkable CTC count of ~ 11 500 CTC/7.5 mL WB at her therapy discontinuation 49 days post‐treatment initiation [25] (Fig. 2, Patient #26).

Although we only sequenced a small fraction of this patient’s large number of CTCs at each time point, her *ESR1* p.Y537C‐mutant CTC fraction at baseline (14/18 CTC) and day 49 (16/18 CTC) was fairly constant (Figs 2 and 3B). Furthermore, this case displayed inter‐CTC heterogeneity of the *ESR1* mutation, with a combination of biallelic and monoallelic mutant as well as *ESR1 wild‐type* cancer cells present in circulation. Yet, the relative distributions of these three configurations in CTC also remained stable between time points (3 : 11 : 4 vs. 3 : 13 : 2, respectively), all in the context of a ~ 4‐fold increase in the absolute CTC count (Fig. 3B). In comparison, her *ESR1* p.Y537C‐mutant ctDNA fraction expectedly increased between C1D1 and day 49, by over 9‐fold. These data support a model of progression in this patient that did not involve changes/sweeps in the clonal genomic architecture of her cancer. Rather, it appeared to occur through other potential mechanisms of progression that increased absolute CTC count (and ctDNA mutant fraction) in circulation while preserving the proportions of CTC subclones. Importantly, such details of this cancer’s molecular makeup would have been unresolvable by the composite genomic picture obtained by ctDNA (Fig. 3B).

### 3.5 Intrapatient, inter‐CTC genomic heterogeneity

Circulating tumor cell genomic profiling through scNGS enables elucidation of circulating intrapatient, inter‐CTC genomic heterogeneity with great precision [24]. Similarly to our previous report in another dataset [24], the patient cohort in this study was characterized by considerable intrapatient CTC genomic heterogeneity at baseline. Truncal alterations such as somatic gain‐of‐function mutations in *PIK3CA* (patient #17 and #34) and *c‐KIT* (#20), or deleterious *CDH1* alterations (#26), served as founder mutational ‘backdrops’ to subclonal genomic aberrations likely arising after these truncal, foundational events (Fig. 2). The latter included *ESR1* LBD mutations (#26: monoallelic, biallelic or absent and #20: monoallelic or absent), *ATM* (#20 mono‐ and biallelic), and *FGFR2* (#34 monoallelic or absent, Fig. 2). Unlike founder alterations, subclonal ones displayed considerable intrapatient, inter‐CTC heterogeneity. Interestingly, the genomic pattern observed in patient #26 CTCs (i.e., a lobular BC with an expected E‐cadherin deleterious LOH mutation, accompanied by a subclonal, heterogeneous, mono‐and biallelic *ESR1* LBD mutation, in the context of a one‐copy *TP53* loss, Fig. 2) had been previously observed by us in a lobular BC patient from a different cohort [24].

Intrapatient heterogeneity of CTC genomic landscape between early and late time points was also observed in a few patients. In patient #26 discussed above, CTC carrying the founder *CDH1* indel, but not the *ESR1* mutation, were present at baseline (Fig. 2, #26, Baseline, CTC#: R10, R11, R17, Fig. 3B). These *ESR1 wild‐type* cells are likely remnants of a more primordial tumor state before acquisition of *ESR1* mutation due to AI selective pressure at some point during the patient’s cancer history. Interestingly, however, this *CDH1*‐mutant ‘primitive’ clone was not present at discontinuation, at which time only cells containing both *CDH1* and *ESR1* mutations were observed (Fig. 2, #26 Discontinuation, Fig. 3B). This could conceivably result from continued positive selection of the *ESR1*‐mutant subclone over the ‘primitive’ clone.

Likewise, in patient #34, the discontinuation sample at +169 days showed the presence of one CTC that did not contain that tumor’s dominant *PIK3CA* mutation, but instead harbored a hotspot *IDH1* mutation (Fig. 2, #34, Discontinuation, CTC#: E6). This mutation was not identified in any CTC at baseline. However, this CTC did harbor the *FGFR2* mutation seen in one baseline and two discontinuation CTC, but not the *PIK3CA* mutation observed in all five baseline CTC and in 11 of 13 CTC at progression.

In addition to clonal and subclonal findings, a considerable number of alterations (37 of 67, 55%) were detected, classified as known cancer drivers, but harbored privately by one individual cell each. These included deleterious mutations in *BRCA2* (#17)*, TSC1* (#26, #35) as well as oncogenic ‘hotspot’ mutations in genes such as *SF3B1* (#34)*, NRAS* (#6), and *SMO* (#35) (Fig. 2 and Fig. S2; *BRCA2*, *SF3B1,* and some other private mutations are not shown in figures. They are listed in Table S4. A list of targeted genes is shown in Table S1). While it is unlikely that these private genomic events drove the bulk of disease burden at the time of collection (and it cannot be excluded that a small fraction of them may potentially be WGA artifacts), it is conceivable that at least some of them may serve as stand‐by ‘raw material’ for tumor evolution, only to become dominant drivers if they confer an advantageous phenotype when exposed to future selective pressures.

### 3.6 Therapeutic targets identified by CTC genomic profiling

CtDNA has been successfully employed in cancer precision medicine. As shown above for *ESR1* mutations, CTC scNGS can faithfully replicate ctDNA sequencing for precision oncology biomarker detection. Importantly, it may provide additional clinically relevant information to complement ctDNA findings. To evaluate the ability to find CTC genomic biomarkers predictive of response to targeted therapies, we analyzed truncal and subclonal (present in > 1 CTC) putative driver alterations in this patient cohort. We developed a novel, CTC liquid biopsy‐based platform, MI‐CTCseq, that not only detects actionable alterations, but also accounts for the clonal identity and clonal predominance of the alterations as well as their zygosity. Mutations and copy number alterations were first classified according to four treatment evidence levels, or tiers, based on commonly used reporting guidelines [30, 38]: Tier 1—FDA‐approved treatments for the current indication; Tier 2—FDA‐approved drugs, recommended for off‐label use by practice guidelines in the current or other cancer indications; Tier 3—investigated in currently enrolling, targeted intervention clinical trials for any cancer type; or Tier 4—for which strong preclinical evidence or biological plausibility exists.

Given the importance of the predominance of an alteration for predicting the extent of response to targeted therapy within a genomically heterogeneous tumor [12], we then refined the MI‐CTCseq classification system by assigning alterations into clonality (CL) subtiers A, B, and C based on whether they were present in ≥ 80%, 50–80%, or < 50% of CTC, respectively. Lastly, given CTC’s exquisite ability to unambiguously determine the homo‐ vs. heterozygous status of a mutation/deletion (compared to bulk tissue or ctDNA sequencing), we enhanced MI‐CTCseq by reporting the precise zygosity of targetable mutations/deletions and losses of heterozygosity (LOH). This is critical in determining one‐copy vs. total deletion or LOH in actionable tumor suppressor such as *BRCA1*, *BRCA2*, and *PALB2*, which are targetable only when inactivated biallelically [42]. The final category assignment for each patient was based on the alteration with the best evidence level (i.e., (a) highest priority tier, (b) highest clonal fraction, and (c) homozygosity/complete deletion) if more than one actionable alteration was present.

Eight of 11 evaluable patients (73%) met MI‐CTCseq criteria for harboring at least one potentially tumor‐driving alteration belonging to Tiers 1–3, with all but one case having > 1 such alterations. These were present in at least one of nine different altered genes. No patients had Tier 4 alterations and the remaining three patients harbored no alterations with any level of actionability evidence. The two (18%) patients with Tier 1 alterations belonged to subtiers CL_A_ and CL_B_, that is, with the alteration present in over 50% of cells and thus likely to respond to a considerable extent to the corresponding targeted therapy. A third patient (9%) had Tier 2‐CL_A_ and five others (45%) had Tier 3 (CL_A_ and _B_) alterations, one of them being homozygous (Table 1).

**Table 1:**
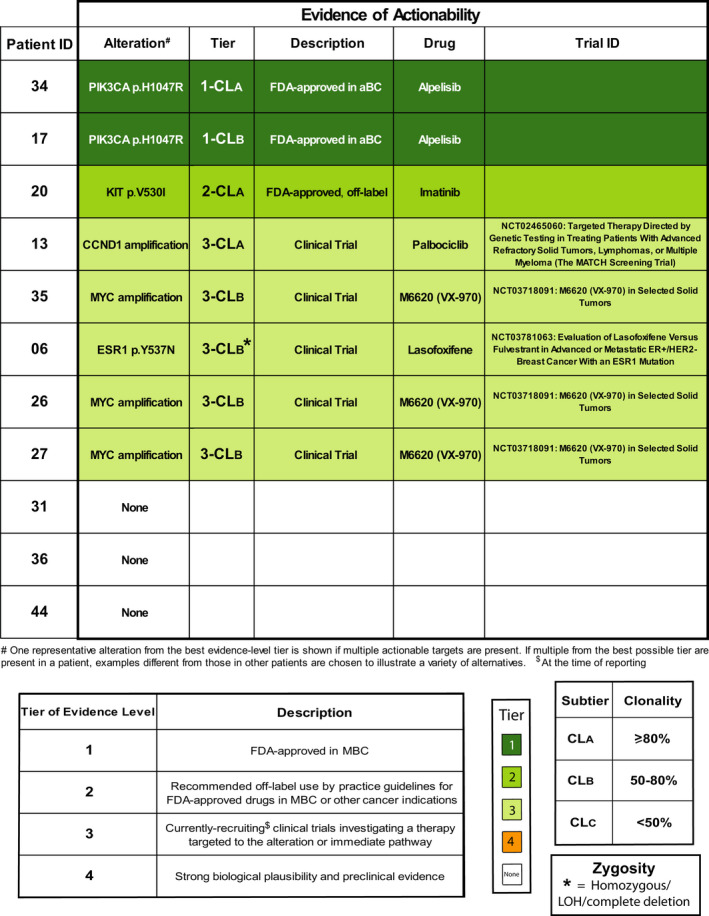
Targetable alterations identified in CTC via the MI‐CTCseq platform. CL, clonality; CTC, circulating tumor cells; MBC, metastatic breast cancer.

The Tier 1 alterations in both patients (#34 CL_A_ and #17 CL_B_) consisted of gain‐of‐function *PIK3CA* mutations for which the targeted inhibitor alpelisib is FDA‐approved in ER‐POS, HER2 NEG breast cancer in conjunction with fulvestrant [43]. Tier 2 alterations detected were gain‐of‐function mutations in *FGFR2* and *c‐KIT*, the latter being the best evidence‐level mutation in the one Tier 2‐CL_A_ patient (#20, Table 1, Fig. 2, Fig. S2). Drugs targeting these two mutant proteins, including erdafitinib and imatinib, respectively, have been approved by the FDA for use in cancers such as urothelial carcinoma and gastrointestinal stromal tumors (GIST), respectively. Tier 3 alterations included amplifications in *MYC* (#26, #27, #35, all in subtier B) and *CCND1* (#13, subtier A), actively investigated in clinical trials. Assignment for the three patients without actionable alterations was potentially affected by the low numbers of CTC recovered for each (Patients #31, #36, and #44, Fig. S2, Table 1).

Taken together, these data support examination of the CTC genomic landscape as a non‐invasive source of actionable biomarkers for precision guided treatment in patients with elevated CTC, similarly to ctDNA. Importantly however, our novel MI‐CTCseq platform, can define the precise CTC subclonal composition in each patient allowing prediction of the subclone likely to respond to the matched therapy and tracking of its clinically relevant fluctuations. Furthermore, we show that our platform can rank actionable alterations based on their subclonal dominance to potentially predict the extent of response. Mutation variant fraction in ctDNA instead is dependent on the tumor burden, tumor DNA shedding ability, content of normal DNA in plasma, etc. Moreover, our CTC‐based system can unambiguously determine the alteration zygosity, important in actionable tumor suppressors, which cannot be reliably accomplished by ctDNA. Lastly, although not examined here, CTCs can provide phenotypic information important for immunotherapy and other biomarkers. Overall, these data support the continued exploration of CTC liquid biopsy‐guided precision/immuno‐oncology to complement ctDNA and tissue analysis.

## 4 Discussion

In this study, we profiled individual CTC genomes prior to therapy initiation and at different therapy time points in 12 patients enrolled in a prospective phase I clinical trial investigating oral SERD AZD9496. We obtained high‐quality genomic information from 123 CTC, detecting 67 putative driver mutations, indels, and high‐level copy number alterations, 45% of which were present in > 1 CTC within individual patients. CTC data were perfectly concordant with concurrent ctDNA ddPCR for *ESR1* LBD mutations. We also analyzed CTC’s ability to enable precision medicine genomic biomarker detection whereby 73% of patients harbored approved or investigational targetable alterations. The therapeutically predictive ability of a genomic biomarker is related to the clonal dominance of that alteration in the context of a genomically heterogeneous tumor [12, 44]. Herein, we propose a novel CTC‐based individualized, precision oncology platform, MI‐CTCseq that factors in the clonal dominance of a targetable genomic event in the circulation exploiting the exquisite ability of CTC to precisely define the tumor clonal architecture. Further, our system incorporates unambiguous identification of alteration zygosity, a feature with important precision medicine implications. This is especially imperative in determining one‐copy vs. total deletion or LOH of actionable tumor suppressors such as *BRCA1*, *BRCA2*, *PALB2,* and others for which biallelic inactivation would make them eligible for targeted therapy [42].

In this cohort, we again observed considerable intrapatient, inter‐CTC genomic heterogeneity at baseline that in some instances became more complex at later time points. We posit that ctDNA, or even scNGS of a localized tissue biopsy would likely be unable to resolve the details of a complex, rapidly evolving disease like cancer. Bulk sequencing approaches such as tissue biopsy and ctDNA have a partial (and often low) tumor content. Thus, unlike CTC, they provide only a composite picture of tumor genomic landscape [45] with often ambiguous determination of subclonality and zygosity. This can over‐rely on rather convoluted analyses of variant read frequencies observed within a tumor‐normal mixture [46]. Our flexible method profiles individual CTCs, but also allows for pooling of multiple CTC into a single sample if simple knowledge of the combined genomic alterations in circulation is needed [24, 47]. Furthermore, although not investigated here, CTC genomic profiling, with its perfect tumor content, is particularly suited to the non‐invasive determination of tumor mutation burden (TMB) and microsatellite instability (MSI), two approved checkpoint inhibitor immunotherapy predictive biomarkers [48, 49], with potentially higher accuracy than currently reported by ctDNA, especially for low tumor content samples [10, 11, 50].

Multiregion sequencing of tumor tissue has confirmed the long‐postulated intratumor genomic heterogeneity [51, 52]. Previous work from other investigators and our group has also revealed this heterogeneity being reflected in CTC [13, 24, 53, 54, 55, 56]. We and others have reported that the CTC genomic landscape is generally, but not perfectly concordant to that of matched tissue samples [24, 53, 54, 57]. CTC *ESR1* mutational heterogeneity [56] and CTC genomic relationship to metastatic and primary tissue [53, 54] have been documented primarily in breast and prostate cancer. In general, intratumor heterogeneity is commonly recognized as fueling tumor evolution resulting in therapy resistance and ultimately death [12, 44, 58, 59, 60]. Indeed, the recognition of cancer heterogeneity was the basis for introduction of combination therapies, which resulted in the first cures and prolongation of survival in human cancers [61]. Assaying that heterogeneity and detecting predictive genomic biomarkers in a non‐invasive, longitudinal, high‐precision method as with CTC scNGS, will potentially allow the clinician to ‘keep‐up’ with a changing disease and adjust precision treatment accordingly to extend survival. For example, the investigation of combination precision therapy, as is currently being investigated in the NCI‐ComboMATCH/EAY191 trial being conducted in North America, represents one first step toward that future paradigm.

Our study has some important limitations. First, our limited patient size (*n* = 12) precludes the discovery of generalizing principles of MBC CTC genomics. Additionally, our time points were relatively closely spaced to fully capture long‐term disease evolution. Additional studies in larger ER POS prospective cohorts with more informative time points remain a focus of our current work. Further, CTC scNGS, with multiple sequenced samples required per patient, is substantially costlier compared to one ctDNA or tissue sample, even though the sequencing depth required per cell can be lower, especially compared to ctDNA.

Another important limitation lies in the fact that CTC capture from ~ 7.5 mL WB can be a low sensitivity approach compared to ctDNA, since only 54% of MBC patients have ≥ 5 CTC/7.5 mL WB [62, 63]. Thus, analyses do not include all patients and can be skewed toward nonresponding, progressing cases with abundant CTC. This concern is further compounded by rejection of some CTC by WGA and scNGS quality filters [64, 65]. The ability to delineate tumor heterogeneity and subclonal makeup is especially limited in patients with low CTC counts making collection and processing of additional 7.5‐mL blood tubes necessary. To address these limitations, we have also recently reported on a mini‐cytopheresis device linked to a short‐term, in‐dwelling, intravascular, dual‐dual lumen catheter system that permits interrogation of larger blood volumes over longer time periods [66], much as a Holter monitor improves analysis of cardiac arrhythmias over a simple electrocardiogram. Such a system should greatly increase CTC capture in each patient and expand the proportion of CTC‐positive patients as has been reported using apheresis‐based methods [54, 67, 68]. It is worth mentioning that ctDNA liquid biopsy also has imperfect sensitivity, with many patients having low ctDNA content (e.g., 43% of advanced non‐small cell lung cancer has < 2% ctDNA tumor content [69]). And obviously, detection of intrapatient heterogeneity in ctDNA as a bulk sample of total tumor DNA in circulation is quite challenging even with high tumor content. Tissue samples for that matter, also have imperfect sensitivity for precision oncology purposes and represent only one area of one lesion. We thus envision CTC sequencing as a method with additional features and specific advantages that could *complement* ctDNA and tissue in many patients. Other limitations of our approach include use of restriction digestion‐based WGA [28, 29], which causes loss of a portion of sequencing panel coverage breadth, an imprecise variant fraction for heterozygous mutations, and imprecise determination of low‐level copy gains/losses in individual cells.

## 5 Conclusion

Comprehensive individual CTC genomic analysis of fresh or archived cells is a feasible way to assess tumor heterogeneity, detect existing and novel features of actionable precision oncology biomarkers, and resolve complex tumor genomic evolution non‐invasively, longitudinally and with great clarity. This may represent a useful approach that complements ctDNA‐based liquid biopsy for elucidating tumor biology and impacting clinical decision making.

## Conflicts of interest

CP received research funding from AstraZeneca, the developer of AZ9496, to conduct this work. She also received research travel reimbursement and research funding from Menarini Silicon Biosystems, the owner of CellSearch and DEPArray. CP previously received research funding from Pfizer, outside the submitted work. CP is currently an employee of EISAI, Inc., but this work is unrelated to her current employment.

DFH reports research financial support related to this study and provided to his institution during conduct and analysis of this study from Menarini/Silicon BioSystems, the manufacturer of CellSearch®. DFH also reports that his institution holds a patent regarding circulating tumor cell characterization for which DFH is the named investigator that was licensed to Menarini Silicon Biosystems and from which both received annual royalties, ending in January 2021. He also reports research support from AstraZeneca, the sponsor of the trial from which the specimens were collected. DFH reports support unrelated to this study but provided to his institution during conduct and analysis of this study from Eli Lilly Company, Merrimack Pharmaceuticals, Inc. (Parexel Intl Corp), Veridex and Janssen Diagnostics (Johnson & Johnson), Pfizer, and Puma Biotechnology, Inc. (subcontract Wash Univ St. Louis to Univ Mich). DFH reports personal income related to consulting or advisory board activities from BioVeca, Cellworks, Cepheid, EPIC Sciences, Freenome, Guardant, L‐Nutra, Oncocyte, Macrogenics, Predictus BioSciences, Salutogenic Innovations, Turnstone Biologics, and Tempus. DFH reports personally held stock options from InBiomotion.

DFH has received research funding from AstraZeneca. SAT is an employee and equity holder in Strata Oncology; research funding has been received from AstraZeneca, Astex Pharmaceuticals, Bioven, Amgen, Carrick Therapeutics, Merck AG, Taiho Oncology, GSK, Bayer, Boehringer Ingelheim, Roche, BMS, Novartis, Celgene, Epigene Therapeutics Inc., Angle PLC, Menarini, Clearbridge Biomedics, Thermo Fisher Scientific, Neomed Therapeutics. CD has received honoraria for consultancy/advisory boards from Biocartis, Merck, and AstraZeneca. The other authors declare no potential conflicts of interest. [Correction added on 22 January 2022, after first online publication: the Conflicts of interest section has been corrected to add a more detailed statement for DFH in this version.]

## Author contributions

AKC contributed to conceptualization, design, experiments, analysis, and writing; EMD contributed to experiments and analysis; EPD contributed to experiments; KH, CJL, and CM contributed to analysis; JP, KB, EK, and KA contributed to experiments; GS contributed to conceptualization and design; DC contributed to conceptualization and design; THC, TK, JL, GM, VR, EAH, JCB, NS, and VS contributed to experiments; AA, RB, EH, SAI, KJ, and MRP contributed as clinical trial investigators; CD, SAT, and AMU contributed to conceptualization and design; DFH and CP contributed to conceptualization, design, and analysis. All authors have reviewed the manuscript.

### Peer Review

The peer review history for this article is available at https://publons.com/publon/10.1002/1878‐0261.13150.

## Supporting information


**Fig. S1.** Short‐term storage in glycerol at ‐20oC vs. CellSearch® media at 4oC yields comparable scNGS quality.
**Fig. S2.** Integrative heatmap of putative driver genomic alterations detected by CTC scNGS and ctDNA ddPCR.
**Table S1.** List of genes targeted in the panel used in this study.
**Table S2.** CTC count by storage conditions and time.
**Table S3.** Sequencing parameters for scNGS.
**Table S4.** ScNGS somatic mutational calls for CTCs passing NGS quality filters.

## Data Availability

The raw data that support the findings presented in this work are fully available upon request to the corresponding author. The data are not publicly available due to patient privacy or ethical restrictions.
