# Supplementary material for: Serial monitoring of genomic alterations in circulating tumor cells of ER‐positive/HER2‐negative advanced breast cancer: feasibility of precision oncology biomarker detection
Source: Mol Oncol. 2021 Dec 20;16(10):1969–85. doi: 10.1002/1878-0261.13150 (PMC9120891; doi:10.1002/1878-0261.13150)
Supplement: Supplementary file 1 — Fig. S1. Short‐term storage in glycerol at ‐20oC vs. CellSearch® media at 4oC yields comparable scNGS quality. Fig. S2. Integrative heatmap of putative driver genomic alterations detected by CTC scNGS and ctDNA ddPCR. Table S1. List of genes targeted in the panel used in this study. Table S2. CTC count by storage conditions and time. Table S3. Sequencing parameters for scNGS. Table S4. ScNGS somatic mutational calls for CTCs passing NGS quality filters. [file MOL2-16-1969-s001.pdf]

Supplementary Materials

Supplementary Results

Supplementary Figure Legends

Supplementary Table Legends

Figure S1. Short-term storage in glycerol at -20ºC vs. CellSearch® media at 4ºC yields comparable scNGS quality.

Figure S2. Integrative heatmap of putative driver genomic alterations detected by CTC scNGS and ctDNA ddPCR.

Table S1. List of genes targeted in the panel used in this study.

Table S2. CTC count by storage conditions and time.

Table S3. Sequencing parameters for scNGS.

Table S4. ScNGS somatic mutational calls for CTCs passing NGS quality filters.

Supplementary Results

CTC storage conditions

During the initial enrollment period, the CTC were stored in the CellSearch® cartridges at 4ºC without additives. However, during enrollment, storage of CTC by removing from CellSearch® cartridges and placing in glycerol at -20ºC for up to two years was reported to be equivalent to fresh cells for DNA isolation and downstream analysis[3]. Therefore, CTC from 8 and 4 of the 12 eligible patients who had ≥5 CTC/7.5 mL WB were stored in cartridges at 4ºC or glycerol at -20ºC, respectively. Although glycerol cells were stored on average for almost twice as long as cartridge cells (46 vs. 24 months), they yielded high sequencing quality cells at a higher rate (100% vs. 86% of CTCs passing scNGS criteria, respectively) although the difference was not statistically significant (*p*-value 0.22, **Table S1**). No difference between the two storage conditions was observed in scNGS quality parameters and identified genomic alterations for MDA-MB-231 cells stored in cartridge vs. glycerol for the short-term (1-4 months, **Figure S1**). Taken together, these data suggest that glycerol at -20ºC may be a preferable long-term storage method for downstream CTC genomic analysis.

Supplementary References

1. Allard WJ, Matera J, Miller MC, et al: Tumor cells circulate in the peripheral blood of all major carcinomas but not in healthy subjects or patients with nonmalignant diseases. Clin Cancer Res 10:6897-904, 2004
2. Paoletti C, Schiavon G, Dolce EM, et al: Circulating Biomarkers and Resistance to Endocrine Therapy in Metastatic Breast Cancers: Correlative Results from AZD9496 Oral SERD Phase I Trial. Clin Cancer Res 24:5860-5872, 2018
3. Mesquita B, Rothwell DG, Burt DJ, et al: Molecular analysis of single circulating tumour cells following long-term storage of clinical samples. Mol Oncol 11:1687-1697, 2017
4. Paoletti C, Cani AK, Larios JM, et al: Comprehensive Mutation and Copy Number Profiling in Archived Circulating Breast Cancer Tumor Cells Documents Heterogeneous Resistance Mechanisms. Cancer Res 78:1110-1122, 2018
5. Peeters DJ, De Laere B, Van den Eynden GG, et al: Semiautomated isolation and molecular characterisation of single or highly purified tumour cells from CellSearch enriched blood samples using dielectrophoretic cell sorting. Br J Cancer 108:1358-67, 2013
6. Polzer B, Medoro G, Pasch S, et al: Molecular profiling of single circulating tumor cells with diagnostic intention. EMBO Mol Med 6:1371-86, 2014
7. Conley BA, Doroshow JH: Molecular analysis for therapy choice: NCI MATCH. Semin Oncol 41:297-9, 2014
8. Hovelson DH, McDaniel AS, Cani AK, et al: Development and validation of a scalable next-generation sequencing system for assessing relevant somatic variants in solid tumors. Neoplasia 17:385-99, 2015
9. Cani AK, Hovelson DH, McDaniel AS, et al: Next-Gen Sequencing Exposes Frequent MED12 Mutations and Actionable Therapeutic Targets in Phyllodes Tumors. Mol Cancer Res 13:613-9, 2015
10. Warrick JI, Hovelson DH, Amin A, et al: Tumor evolution and progression in multifocal and paired non-invasive/invasive urothelial carcinoma. Virchows Arch 466:297-311, 2015
11. Grasso C, Butler T, Rhodes K, et al: Assessing copy number alterations in targeted, amplicon-based next-generation sequencing data. J Mol Diagn 17:53-63, 2015

Figure S1

A

MDA-MB-231 Cells	CellSearch Cartridge 4°C	Glycerol -20°C
Storage (months)	*1 - 4*	*1 - 3*
CTCs analyzed (#)	6	6
WGA DNA amount (ng/ul) Mean (STDEV)	23.8 (3.3)	21.2 (4.5)
WGA high-quality CTCs obtained	100%	100%
scNGS high-quality CTCs (%)	100%	100%
scNGS uniformity Mean (STDEV)	40.6% (2.0%)	41.1% (2.3%)

B

MDA-MB-231 Cells	CellSearch Cartridge (4°C)				Glycerol (-20°C)			
Alteration \ Cell ID	B6	E3	F3	E6*	H3	C3	G3	B3*
TP53 p.R280K	1.00	1.00	1.00	0.88	1.00	1.00	1.00	1.00

C

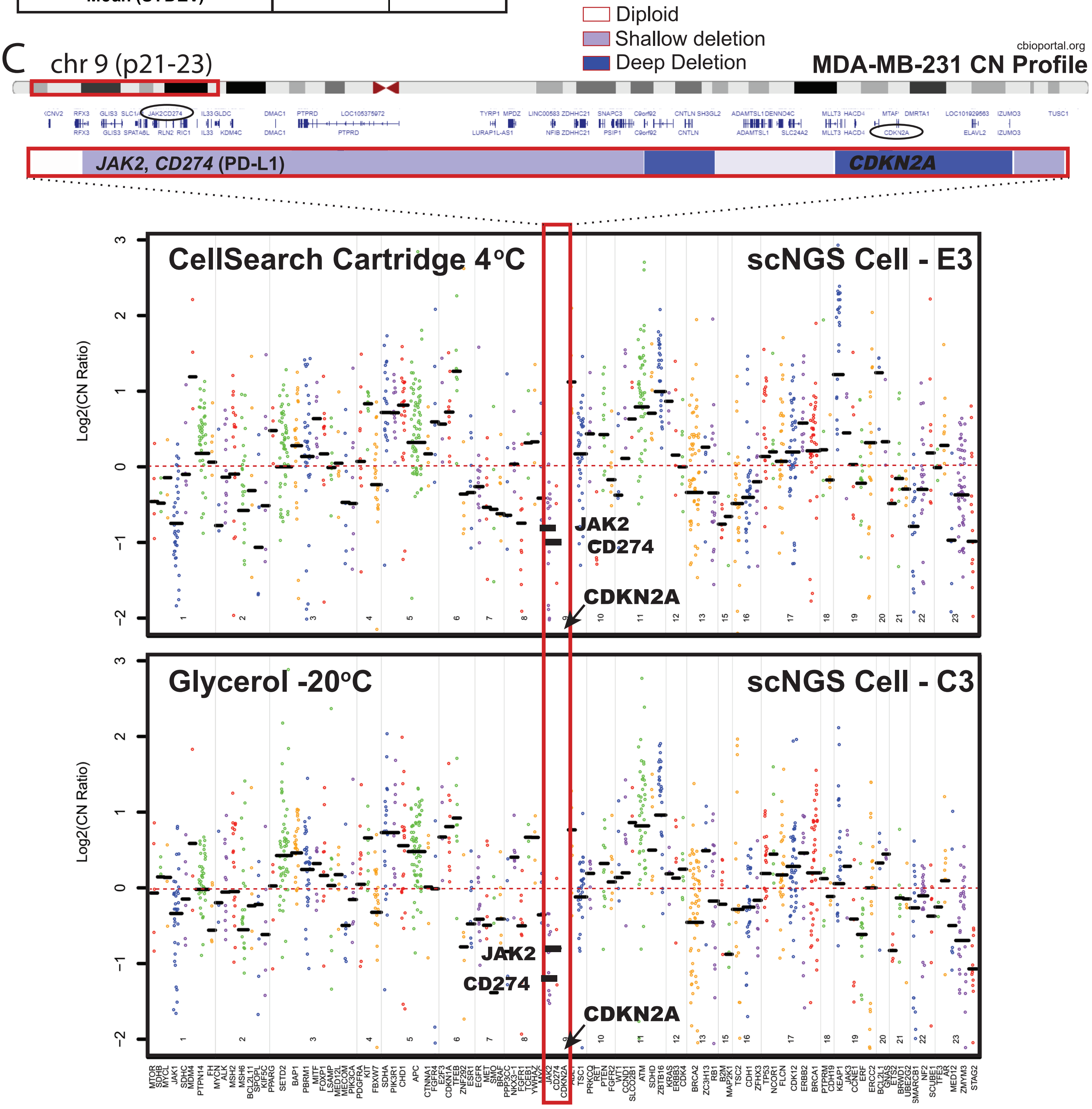

Supplementary Figure Legends

Figure S1. Short-term storage in glycerol at -20ºC vs. CellSearch® media at 4ºC yields comparable scNGS quality.

MDA-MB-231 cells were grown, washed, spiked into healthy donor blood and processed with CellSearch® to mimic patient sample processing. After storage in the two different conditions for 1-4 months, individual DEPArray™-recovered high WGA quality cells underwent scNGS.

A) DNA recovery, success rate and select sequencing quality parameters are tabulated showing comparably high quality. Uniformity is defined as the percentage of target bases covered by at least 0.2x the average read depth.

B) scNGS of recovered individual MDA-MB-231 cells successfully detected the known *TP53* p.R280K "hotspot" mutation in all 8 cells. Each column represents one cell. *denotes cells of slightly lower quality than the rest (Cell E6 uniformity = 24%. Cutoff = 20%, common uniformity observed at >40%). Colored boxes indicate mutation presence with dark and light green representing homo- and heterozygous mutations, respectively. Numbers inside colored boxes represent the variant-containing read fraction.

C) scNGS of recovered individual MDA-MB-231 cells successfully detected the known copy-number alterations targeted by our panel in all 8 cells from both storage conditions. *Top:* known MDA-MB-231 copy-number alterations in ch9 (p21-23) region (cbioportal.org) include a complete deletion of *CDKN2A* (dark blue) accompanied by one-copy deletion of nearby genes *JAK2* and *CD274* (PD-L1, light blue). *Bottom:* representative scNGS copy-number plots for one cell from each of the two storage

conditions accurately identifies deletions of the three genes and their precise copy levels. Panel copy-number genes in chromosomes 1 through X are labeled at the bottom. Colored circles represent individual amplicons and genes are denoted by a collection of same-color amplicons. A gene-level copy number estimate (black bar) is calculated as the coverage-weighted mean of individual amplicons. Plotted is the GC-content-normalized copy-number ratio of the cancer sample to the average of a composite of 10 normal white blood cells from different patients sequenced with the same panel, for normalization. Plots are in log 2 scale (Log2CN ratio) on the y-axis. Relevant genes are labeled on the plots, with the *CDKN2A* deep deletion being below the bottom of the chart (arrow).

Figure S2

Patient #35

~~F~~ AI

Baseline: 76 CTC/7.5 mL WB — Discontinuation (+45d): 217 CTC/7.5 mL WB

		Baseline CTCs (scNGS)	Baseline ctDNA	Discontinuation CTCs (scNGS)									Discontinuation ctDNA
		R7	ddPCR	R19	R12	R9	R2	R11	R4	R16	R1	R3	ddPCR
Mutations / Indels	ESR1 p.Y537S		Y537S	0.45	0.27	0.89	0.37						Y537S
	SMO p.C469F		NA			0.54			NC				NA
	TSC1 p.G1035fs		NA				0.26					NC	NA
CNAs	MYC	5.94		6.39	6.06			5.20	8.59	4.67			
	PIK3R1	0.25		4.44		5.25		5.34	4.96	8.58	5.65	6.03	

Patient #13

F AI

C1D15: 46 CTC/7.5 mL WB

		CTCs (scNGS)		ctDNA
		R3	R5	ddPCR
Mut./Ind.	SDHA p.Y417X	0.25		NA
CNAs	CCND1	8.35	11.4	
	MYC	5.05	7.42	

Patient #06

F AI

Baseline: 13 CTC/7.5 mL WB — Disc. (+56d): 31 CTC/7.5 mL WB

		Baseline CTCs (scNGS)			Baseline ctDNA	Disc. CTCs (scNGS)	Disc. ctDNA
		D3	A3	C3	ddPCR	C3	ddPCR
Mutations / Indels	ESR1 p.Y537N	1.00				1.00	Y537N
	NRAS p.A83V	0.23			NA		NA
	NKX3-1 p.E107X		0.23		NA		NA
CNAs	MYC	4.76				4.75	
	GNAS	5.07				4.43	
	CDKN2A	0.00				0.00	

Patient #31

F AI

Baseline: 15 CTC/7.5 mL WB

	Alter. \ ID	CTCs (scNGS) R7	R4	R6	ctDNA ddPCR
Mutations / Indels	PIK3CA p.H1047R	0.80			NA
	ESR1 p.D538G	0.45			D535G
CNAs	KRAS	4.15	5.81		

Legend

- Homozygous Mutation / Indel (Variant Fraction) (1.00)
- Heterozygous Mutation / Indel (Variant Fraction) (0.50)
- CN gain (copy # estimate) (6.00)
- CN loss (copy # estimate) (0.05)
- ESR1 ddPCR+ (aminoacid substitution) (Y537S)
- Not assessed (no NGS coverage) (NC)
- No alteration

Prior Therapy

- F AI: Fulvestrant and Aromatase Inhibitor
- ~~F~~ AI: Aromatase Inhibitor No Fulvestrant

Patient #27

F AI

Baseline: 192 CTC/7.5 mL WB — C1D15: 166 CTC/7.5 mL WB

		Baseline CTCs (scNGS)		Baseline ctDNA	C1D15 CTCs	C1D15 ctDNA
		R2	R8	ddPCR	R10*	ddPCR
Mut./Ind.	SETD2 p.V774fsins			NA	0.41	NA
CNAs	MYC	7.21	5.66		0.06	

Patient #36

F AI

Baseline: 14 CTC/7.5 mL WB

		CTCs (scNGS)	ctDNA
		R5	ddPCR
Mut./Ind.	None		
CNAs	None		

Patient #44

F AI

Baseline: 16 CTC/7.5 mL WB

		CTCs (scNGS)		ctDNA
		R2*	R7*	ddPCR
Mut./Ind.	None			
CNAs	None			

Figure S2. Integrative heatmap of putative driver genomic alterations detected by CTC scNGS and ctDNA ddPCR.

Comprehensive genomic analysis of individual CTCs from the remaining seven evaluable patients not shown in Figure 2 is presented. For each patient, total CTC count at each time point is shown. Columns represent individual CTCs. White boxes indicate adequate coverage and absence of the variant. For mutations/indels (top of each table) colored boxes indicate mutation presence, with dark and light green representing homo- and heterozygous mutations, respectively. Numbers inside colored boxes represent the variant read fraction. Grey "NC" boxes indicate no NGS coverage for that position. Mutations private to single cells are shown only in select cases. For CNAs (bottom of each table), estimated copy number is calculated back from the log2(Tumor/Normal Copy Ratio) value. High-confidence, high-level copy changes (<0.25 or >4.0 estimated copies) are shown, with red and blue representing amplifications and deletions, respectively. Only high-level CNAs present in >1 patient CTC are shown. *Cells with suboptimal CN data. DdPCR *ESR1* LBD mutation presence/absence and amino acid change are shown at the right end of each table. Later time points for ddPCR consist of C1D15 samples only. Not all CTC harboring only single private alterations, or epithelial cells without alterations, are shown.

Table S1

Genes Targeted						
ABL1	CDK12	ETV1	KEAP1	MTOR	PTPRM	SMO
AKT1	CDK4	FBXW7	KIF5C	MYC	RAC1	SPOP
ALK	CDK6	FGFR1	KIT	MYCL	RAF1	SPOPL
APC	CDKN1A	FGFR2	KLF3	MYCN	RB1	STAG2
AR	CDKN1B	FGFR3	KLK2/3	NCOR1	RET	TCEB1
ARAF	CDKN2A	FGFR4	KLK6	NF2	RHOA	TFE3
ATM	CHD1	FH	KRAS	NFE2L2	ROS1	TFEB
B2M	CTNNA1	FLCN	LSAMP	NKX3-1	RXRA	TP53
BAP1	CTNNB1	FOXA1	MAP2K1	NRAS	RYBP	TSC1
BCL2L1	DDR2	FOXP1	MAP2K2	NTRK1	SAV1	TSC2
BCL2L11	DEK	GNA11	MAP2K7	PBRM1	SCUBE1	UBE2G2
BRAF	E2F3	GNAQ	MDM2	PDGFRA	SDHA	WT1
BRCA1	EGFR	GNAS	MDM4	PIK3CA	SDHB	YWHAZ
BRCA2	ERBB2	HRAS	MECOM	PIK3CB	SDHC	ZBTB16
BRWD1	ERBB3	HSD3B1	MED12	PIK3R1	SDHD	ZC3H13
CCND1	ERBB4	IDH1	MED12L	PPARG	SETD2	ZFHX3
CCNE1	ERCC2	IDH2	MET	PPP3CC	SF3B1	ZMYM3
CD274	ERF	JAK1	MITF	PRKCQ	SLCO2B1	ZNF292
CDH1	ESR1	JAK2	MSH2	PTEN	SMARCA4	
CDH19	ETS2	JAK3	MSH6	PTPN14	SMARCB1	

List of genes targeted in the panel used in this study. Not all genes have their entire coding regions fully targeted.

Table S1. List of genes targeted in the panel used in this study.

The 138 genes targeted in the panel are listed alphabetically. Not all genes have their entire coding regions fully targeted.

Table S2

	CellSearch Cartridge 4°C	Glycerol -20°C
Mean storage (months)	*24*	*46*
**CTCs recovered (#)**	185	190
**WGA high-quality CTCs recovered (#)**	72	61
**scNGS high-q. CTCs recovered (#)**	62	61
**scNGS high-q. CTCs recovered (% of high-q. WGA)**	**86.1%**	**100.0%**

CTC count by storage conditions and time. CTCs were stored either in their original CellSearch® cartridges at 4°C for an average of 24 months or in glycerol at -20°C for an average of 46 months. Number of CTCs, high-quality WGA CTCs and high-quality scNGS CTCs recovered as well as the % recovery of the latter from high-quality WGA CTCs are shown.

Supplementary Table Legends

Table S2. CTC count by storage conditions and time. CTCs were stored either in their original CellSearch® cartridges at 4ºC for an average of 24 months or in glycerol at -20ºC for an average of 46 months. Number of CTCs, high-quality WGA CTCs and high-quality scNGS CTCs recovered as well as the % recovery of the latter from high-quality WGA CTCs are shown.

Table S3

Sample	Mapped Reads	On-Target Reads	Mean Depth	Uniformity	Mean Read Length (bp)
**Mean (Range)**	**2,932,356** (644,022 - 4,973,416)	**90.5%** (24.1 - 96.2%)	**983x** (47 - 1,711x)	**40.1%** (25.4 - 63.8%)	**141** (99 - 152)

ScNGS of 123 high-quality patient CTCs generated sequencing data with excellent quality parameters. Table shows mean of total reads mapped (to the refererence human genome), on-target reads (mapping to the panel target regions), mean depth of sequencing, uniformity (measuring distribution of reads thorughout the target regions and defined as percentage of reads having at least 0.2x of the mean coverage. Uniformity determination includes all panel amplicons, including those affected by the WGA restriction enzyme treatment). Mean read length in base-pairs (bp) is shown.

Table S3. Sequencing parameters for scNGS. 123 high-quality patient CTCs generated sequencing data with excellent quality parameters. Table shows mean of total reads mapped (to the reference human genome), on-target reads (mapping to the panel target regions), mean depth of sequencing, uniformity (measuring distribution of reads throughout the target regions and defined as percentage of reads having at least 0.2x of the mean coverage), and mean read length in base-pairs (bp).

Table S4

Patient #	Timepoint	CTC ID	Genomic location	Reference allele	Variant allele	Variant fraction	Exonic/Other	Function	Gene	RefSeq ID	Nucleotide position	Amino acid	Putative driver (D) / VUS
06	Screening	D3	chr6:152419922	T	A	1.00	exonic	SNV	ESR1	NM_000125	c.T1609A	p.Y537N	D
06	Screening	A3	chr8:23539120	C	A	0.23	exonic	stopgain	NKX3-1	NM_006167	c.G319T	p.E107X	D
06	Screening	D3	chr1:115256463	G	A	0.23	exonic	SNV	NRAS	NM_002524	c.C248T	p.A83V	D
06	Screening	D3	chr17:7578260	C	T	0.16	exonic	SNV	TP53	NM_000546	c.G589A	p.V197M	D
06	Screening	A3	chrX:70472577	C	A	0.22	exonic	stopgain	ZMYM3	NM_005096	c.G529T	p.E177X	D
06	Discontinuation	C3	chr6:152419922	T	A	1.00	exonic	SNV	ESR1	NM_000125	c.T1609A	p.Y537N	D
13	C1D15	R3	chr5:235445	C	A	0.25	exonic	stopgain	SDHA	NM_004168	c.C1251A	p.Y417X	D
17	Baseline	F3	chr5:112175240	G	C	0.53	exonic	SNV	APC	NM_000038	c.G3949C	p.E1317Q	VUS
17	Screening	D9	chr5:112175240	G	C	0.31	exonic	SNV	APC	NM_000038	c.G3949C	p.E1317Q	VUS
17	Screening	A6	chr5:112176396	G	C	0.50	exonic	SNV	APC	NM_000038	c.G5105C	p.G1702A	VUS
17	Screening	A9	chr5:112176396	G	C	0.50	exonic	SNV	APC	NM_000038	c.G5105C	p.G1702A	VUS
17	Screening	E3	chr5:112176396	G	C	0.20	exonic	SNV	APC	NM_000038	c.G5105C	p.G1702A	VUS
17	Screening	F3	chr5:112176396	G	C	0.42	exonic	SNV	APC	NM_000038	c.G5105C	p.G1702A	VUS
17	Screening	F6	chr5:112176396	G	C	0.54	exonic	SNV	APC	NM_000038	c.G5105C	p.G1702A	VUS
17	Screening	G6	chr5:112176396	G	C	0.65	exonic	SNV	APC	NM_000038	c.G5105C	p.G1702A	VUS
17	Screening	H9	chr5:112176396	G	C	0.56	exonic	SNV	APC	NM_000038	c.G5105C	p.G1702A	VUS
17	Screening	D9	chr3:52437471	AG	A	0.17	exonic	frameshift deletion	BAP1	NM_004656	c.1690delC	p.H563fs	D
17	Screening	F3	chr13:32913203	G	T	0.12	exonic	stopgain	BRCA2	NM_000059	c.G4711T	p.E1571X	D
17	Discontinuation	E3	chr16:68853193	T	TG	1.00	exonic	frameshift insertion	CDH1	NM_004360	c.1577dupG	p.W526fs	D
17	Screening	D9	chr19:45855565	G	A	0.12	exonic	stopgain	ERCC2	NM_000400	c.C2092T	p.Q698X	D
17	Screening	D9	chr4:153332625	C	A	0.10	exonic	stopgain	FBXW7	NM_033632	c.G331T	p.E111X	D
17	Screening	E3	chr15:66729181	A	G	0.10	exonic	SNV	MAP2K1	NM_002755	c.A389G	p.Y130C	D
17	Screening	A6	chr1:11190804	C	T	0.44	exonic	SNV	MTOR	NM_004958	c.G5395A	p.E1799K	D
17	Screening	E3	chr3:178952085	A	G	0.75	exonic	SNV	PIK3CA	NM_006218	c.A3140G	p.H1047R	D
17	Screening	F6	chr3:178952085	A	G	0.55	exonic	SNV	PIK3CA	NM_006218	c.A3140G	p.H1047R	D
17	Screening	G6	chr3:178952085	A	G	0.42	exonic	SNV	PIK3CA	NM_006218	c.A3140G	p.H1047R	D
17	Screening	F3	chr3:178952085	A	G	0.39	exonic	SNV	PIK3CA	NM_006218	c.A3140G	p.H1047R	D
17	Screening	H9	chr3:178952085	A	G	0.33	exonic	SNV	PIK3CA	NM_006218	c.A3140G	p.H1047R	D
17	Screening	A6	chr3:178952085	A	G	0.29	exonic	SNV	PIK3CA	NM_006218	c.A3140G	p.H1047R	D
17	Screening	A9	chr3:178952085	A	G	0.20	exonic	SNV	PIK3CA	NM_006218	c.A3140G	p.H1047R	D
17	Screening	F6	chr10:89692907	A	G	0.14	exonic	SNV	PTEN	NM_000314	c.A391G	p.T131A	D
17	Baseline	F3	chr16:2112558	G	A	0.37	exonic	SNV	TSC2	NM_000548	c.G1318A	p.G440S	VUS
17	Screening	A6	chr16:2112558	G	A	0.51	exonic	SNV	TSC2	NM_000548	c.G1318A	p.G440S	VUS
17	Screening	D9	chr16:2112558	G	A	0.50	exonic	SNV	TSC2	NM_000548	c.G1318A	p.G440S	VUS
17	Screening	F3	chr16:2112558	G	A	0.51	exonic	SNV	TSC2	NM_000548	c.G1318A	p.G440S	VUS
17	Screening	F6	chr16:2112558	G	A	0.57	exonic	SNV	TSC2	NM_000548	c.G1318A	p.G440S	VUS
17	Screening	G6	chr16:2112558	G	A	0.17	exonic	SNV	TSC2	NM_000548	c.G1318A	p.G440S	VUS
17	Screening	A6	chr11:114121123	C	T	0.63	exonic	SNV	ZBTB16	NM_006006	c.C1868T	p.T623M	VUS
17	Screening	A9	chr11:114121123	C	T	0.99	exonic	SNV	ZBTB16	NM_006006	c.C1868T	p.T623M	VUS
17	Screening	E3	chr11:114121123	C	T	0.81	exonic	SNV	ZBTB16	NM_006006	c.C1868T	p.T623M	VUS
17	Screening	F3	chr11:114121123	C	T	1.00	exonic	SNV	ZBTB16	NM_006006	c.C1868T	p.T623M	VUS
17	Screening	F6	chr11:114121123	C	T	0.42	exonic	SNV	ZBTB16	NM_006006	c.C1868T	p.T623M	VUS
17	Screening	G6	chr11:114121123	C	T	0.78	exonic	SNV	ZBTB16	NM_006006	c.C1868T	p.T623M	VUS
17	Screening	H9	chr11:114121123	C	T	0.87	exonic	SNV	ZBTB16	NM_006006	c.C1868T	p.T623M	VUS
17	Discontinuation	A4	chr5:112176396	G	C	0.32	exonic	SNV	APC	NM_000038	c.G5105C	p.G1702A	VUS
17	Discontinuation	C11	chr5:112176396	G	C	0.49	exonic	SNV	APC	NM_000038	c.G5105C	p.G1702A	VUS
17	Discontinuation	G4	chr5:112176396	G	C	0.52	exonic	SNV	APC	NM_000038	c.G5105C	p.G1702A	VUS
17	Discontinuation	H8	chr5:112176396	G	C	0.99	exonic	SNV	APC	NM_000038	c.G5105C	p.G1702A	VUS
17	Discontinuation	A4	chr11:108139278	TTGTTAA	T	0.54	exonic	framepreserving deletion	ATM	NM_000051	c.2780_2785del	p.L928_M929del	VUS
17	Discontinuation	H8	chr11:108139278	TTGTTAA	T	0.26	exonic	framepreserving deletion	ATM	NM_000051	c.2780_2785del	p.L928_M929del	VUS
17	Discontinuation	H8	chr16:68853193	T	TG	1.00	exonic	frameshift insertion	CDH1	NM_004360	c.1577dupG	p.W526fs	D

Patient #	Timepoint	CTC ID	Genomic location	Reference allele	Variant allele	Variant fraction	Exonic/Other	Function	Gene	RefSeq ID	Nucleotide position	Amino acid	Putative driver (D) / VUS
17	Discontinuation	C11	chr16:68853193	T	TG	1.00	exonic	frameshift insertion	CDH1	NM_004360	c.1577dupG	p.W526fs	D
17	Discontinuation	D4	chr5:98212218	ATC	A	0.14	exonic	frameshift deletion	CHD1	NM_001270	c.3281_3282del	p.P1093fs	D
17	Discontinuation	A11	chr17:37880997	G	T	0.11	exonic	SNV	ERBB2	NM_004448	c.G2326T	p.G776C	D
17	Discontinuation	A11	chr19:45857990	TG	T	1.00	exonic	frameshift deletion	ERCC2	NM_000400	c.1663delC	p.G556fs	D
17	Discontinuation	F11	chr11:534291	G	A	0.19	exonic	SNV	HRAS	NM_005343	c.C32T	p.A11V	D
17	Discontinuation	F11	chr3:178938896	ATTAAC	A	0.52	exonic	stopgain	PIK3CA	NM_006218	c.2138_2142del	p.N714X	VUS
17	Discontinuation	A4	chr3:178952085	A	G	0.33	exonic	SNV	PIK3CA	NM_006218	c.A3140G	p.H1047R	D
17	Discontinuation	H8	chr3:178952085	A	G	0.45	exonic	SNV	PIK3CA	NM_006218	c.A3140G	p.H1047R	D
17	Discontinuation	B4	chr3:49412955	A	G	0.11	exonic	SNV	RHOA	NM_001664	c.T68C	p.I23T	D
17	Discontinuation	B11	chr5:236628	C	T	0.97	exonic	SNV	SDHA	NM_004168	c.C1346T	p.A449V	VUS
17	Discontinuation	D4	chr5:236628	C	T	0.10	exonic	SNV	SDHA	NM_004168	c.C1346T	p.A449V	VUS
17	Discontinuation	A11	chr3:47165837	G	A	0.12	exonic	stopgain	SETD2	NM_014159	c.C289T	p.Q97X	D
17	Discontinuation	B4	chr17:7577520	A	G	0.12	exonic	SNV	TP53	NM_000546	c.T761C	p.I254T	D
17	Discontinuation	A4	chr16:2112558	G	A	0.65	exonic	SNV	TSC2	NM_000548	c.G1318A	p.G440S	VUS
17	Discontinuation	C11	chr16:2112558	G	A	0.96	exonic	SNV	TSC2	NM_000548	c.G1318A	p.G440S	VUS
17	Discontinuation	C11	chr11:114121123	C	T	0.63	exonic	SNV	ZBTB16	NM_006006	c.C1868T	p.T623M	VUS
17	Discontinuation	G4	chr11:114121123	C	T	1.00	exonic	SNV	ZBTB16	NM_006006	c.C1868T	p.T623M	VUS
17	Discontinuation	H8	chr11:114121123	C	T	0.65	exonic	SNV	ZBTB16	NM_006006	c.C1868T	p.T623M	VUS
17	Discontinuation	D4	chrX:70462909	A	G	0.14	exonic	SNV	ZMYM3	NM_005096	c.T3458C	p.V1153A	VUS
17	Discontinuation	F8	chrX:70462910	C	T	0.11	exonic	SNV	ZMYM3	NM_005096	c.G3457A	p.V1153M	VUS
20	Discontinuation	F6	chr11:108138003	T	C	1.00	exonic	SNV	ATM	NM_000051	c.T2572C	p.F858L	D
20	Discontinuation	H3	chr11:108138003	T	C	0.67	exonic	SNV	ATM	NM_000051	c.T2572C	p.F858L	D
20	Discontinuation	A9	chr3:52437884	TG	T	0.31	exonic	frameshift deletion	BAP1	NM_004656	c.1277delC	p.K425fs	D
20	Discontinuation	G3	chr17:41197778	A	C	0.14	exonic	SNV	BRCA1	NM_007294	c.T5509G	p.W1837G	D
20	Discontinuation	A3	chr17:41231413	G	T	0.19	exonic	stopgain	BRCA1	NM_007300	c.C4361A	p.S1454X	D
20	Discontinuation	A9	chr12:58145436	T	C	0.12	exonic	SNV	CDK4	NM_000075	c.A65G	p.K22R	D
20	Discontinuation	C6	chr5:98235307	TTTAA	T	0.33	exonic	frameshift deletion	CHD1	NM_001270	c.959_962del	p.L319fs	D
20	Discontinuation	A3	chr19:45868302	C	A	0.30	exonic	stopgain	ERCC2	NM_000400	c.G475T	p.E159X	D
20	Discontinuation	A9	chr6:152419926	A	G	0.43	exonic	SNV	ESR1	NM_000125	c.A1613G	p.D538G	D
20	Discontinuation	B6	chr1:65332647	C	A	0.17	exonic	stopgain	JAK1	NM_002227	c.G892T	p.E298X	D
20	Discontinuation	A3	chr4:55593431	G	A	0.41	exonic	SNV	KIT	NM_000222	c.G1588A	p.V530I	D
20	Discontinuation	A9	chr4:55593431	G	A	0.51	exonic	SNV	KIT	NM_000222	c.G1588A	p.V530I	D
20	Discontinuation	B6	chr4:55593431	G	A	0.77	exonic	SNV	KIT	NM_000222	c.G1588A	p.V530I	D
20	Discontinuation	C6	chr4:55593431	G	A	0.66	exonic	SNV	KIT	NM_000222	c.G1588A	p.V530I	D
20	Discontinuation	G3	chr4:55593431	G	A	0.42	exonic	SNV	KIT	NM_000222	c.G1588A	p.V530I	D
20	Discontinuation	H3	chr4:55593431	G	A	0.45	exonic	SNV	KIT	NM_000222	c.G1588A	p.V530I	D
26	Baseline	R13	chr5:112174286	C	T	0.28	exonic	stopgain	APC	NM_000038	c.C2995T	p.Q999X	D
26	Baseline	R1	chr3:52441321	C	T	0.36	exonic	SNV	BAP1	NM_004656	c.G449A	p.R150H	VUS
26	Baseline	R9	chr13:32912237	G	T	0.30	exonic	stopgain	BRCA2	NM_000059	c.G3745T	p.E1249X	D
26	Baseline	R3	chr13:32936802	G	C	0.16	exonic	SNV	BRCA2	NM_000059	c.G7948C	p.E2650Q	VUS
26	Baseline	R4	chr13:32936802	G	C	0.51	exonic	SNV	BRCA2	NM_000059	c.G7948C	p.E2650Q	VUS
26	Baseline	R5	chr13:32936802	G	C	0.59	exonic	SNV	BRCA2	NM_000059	c.G7948C	p.E2650Q	VUS
26	Baseline	R6	chr13:32936802	G	C	0.87	exonic	SNV	BRCA2	NM_000059	c.G7948C	p.E2650Q	VUS
26	Baseline	R7	chr13:32936802	G	C	0.20	exonic	SNV	BRCA2	NM_000059	c.G7948C	p.E2650Q	VUS
26	Baseline	R8	chr13:32936802	G	C	0.61	exonic	SNV	BRCA2	NM_000059	c.G7948C	p.E2650Q	VUS
26	Baseline	R1	chr13:32936802	G	C	0.51	exonic	SNV	BRCA2	NM_000059	c.G7948C	p.E2650Q	VUS
26	Baseline	R10	chr13:32936802	G	C	0.56	exonic	SNV	BRCA2	NM_000059	c.G7948C	p.E2650Q	VUS
26	Baseline	R11	chr13:32936802	G	C	0.67	exonic	SNV	BRCA2	NM_000059	c.G7948C	p.E2650Q	VUS
26	Baseline	R13	chr13:32936802	G	C	0.75	exonic	SNV	BRCA2	NM_000059	c.G7948C	p.E2650Q	VUS
26	Baseline	R14	chr13:32936802	G	C	0.53	exonic	SNV	BRCA2	NM_000059	c.G7948C	p.E2650Q	VUS
26	Baseline	R15	chr13:32936802	G	C	0.32	exonic	SNV	BRCA2	NM_000059	c.G7948C	p.E2650Q	VUS
26	Baseline	R17	chr13:32936802	G	C	0.75	exonic	SNV	BRCA2	NM_000059	c.G7948C	p.E2650Q	VUS
26	Baseline	R19	chr13:32936802	G	C	0.53	exonic	SNV	BRCA2	NM_000059	c.G7948C	p.E2650Q	VUS
26	Baseline	R2	chr13:32936802	G	C	0.16	exonic	SNV	BRCA2	NM_000059	c.G7948C	p.E2650Q	VUS
26	Baseline	R21	chr13:32936802	G	C	0.16	exonic	SNV	BRCA2	NM_000059	c.G7948C	p.E2650Q	VUS

Patient #	Timepoint	CTC ID	Genomic location	Reference allele	Variant allele	Variant fraction	Exonic/Other	Function	Gene	RefSeq ID	Nucleotide position	Amino acid	Putative driver (D) / VUS
26	Baseline	R3	chr16:68842724	TG	T	1.00	exonic	frameshift deletion	CDH1	NM_004360	c.660delG	p.L220fs	D
26	Baseline	R4	chr16:68842724	TG	T	1.00	exonic	frameshift deletion	CDH1	NM_004360	c.660delG	p.L220fs	D
26	Baseline	R5	chr16:68842724	TG	T	1.00	exonic	frameshift deletion	CDH1	NM_004360	c.660delG	p.L220fs	D
26	Baseline	R6	chr16:68842724	TG	T	1.00	exonic	frameshift deletion	CDH1	NM_004360	c.660delG	p.L220fs	D
26	Baseline	R6	chr16:68842724	TG	T	1.00	exonic	frameshift deletion	CDH1	NM_004360	c.660delG	p.L220fs	D
26	Baseline	R7	chr16:68842724	TG	T	1.00	exonic	frameshift deletion	CDH1	NM_004360	c.660delG	p.L220fs	D
26	Baseline	R8	chr16:68842724	TG	T	1.00	exonic	frameshift deletion	CDH1	NM_004360	c.660delG	p.L220fs	D
26	Baseline	R1	chr16:68842724	TG	T	1.00	exonic	frameshift deletion	CDH1	NM_004360	c.660delG	p.L220fs	D
26	Baseline	R10	chr16:68842724	TG	T	1.00	exonic	frameshift deletion	CDH1	NM_004360	c.660delG	p.L220fs	D
26	Baseline	R11	chr16:68842724	TG	T	1.00	exonic	frameshift deletion	CDH1	NM_004360	c.660delG	p.L220fs	D
26	Baseline	R14	chr16:68842724	TG	T	1.00	exonic	frameshift deletion	CDH1	NM_004360	c.660delG	p.L220fs	D
26	Baseline	R15	chr16:68842724	TG	T	1.00	exonic	frameshift deletion	CDH1	NM_004360	c.660delG	p.L220fs	D
26	Baseline	R17	chr16:68842724	TG	T	1.00	exonic	frameshift deletion	CDH1	NM_004360	c.660delG	p.L220fs	D
26	Baseline	R19	chr16:68842724	TG	T	1.00	exonic	frameshift deletion	CDH1	NM_004360	c.660delG	p.L220fs	D
26	Baseline	R2	chr16:68842724	TG	T	1.00	exonic	frameshift deletion	CDH1	NM_004360	c.660delG	p.L220fs	D
26	Baseline	R14	chr9:21971096	C	G	0.20	exonic	SNV	CDKN2A	NM_000077	c.G262C	p.E88Q	D
26	Baseline	R1	chr19:42753645	G	A	0.31	exonic	stopgain	ERF	NM_006494	c.C619T	p.R207X	D
26	Baseline	R3	chr6:152419923	A	G	0.38	exonic	SNV	ESR1	NM_000125	c.A1610G	p.Y537C	D
26	Baseline	R4	chr6:152419923	A	G	0.46	exonic	SNV	ESR1	NM_000125	c.A1610G	p.Y537C	D
26	Baseline	R5	chr6:152419923	A	G	0.59	exonic	SNV	ESR1	NM_000125	c.A1610G	p.Y537C	D
26	Baseline	R6	chr6:152419923	A	G	0.29	exonic	SNV	ESR1	NM_000125	c.A1610G	p.Y537C	D
26	Baseline	R7	chr6:152419923	A	G	1.00	exonic	SNV	ESR1	NM_000125	c.A1610G	p.Y537C	D
26	Baseline	R8	chr6:152419923	A	G	0.38	exonic	SNV	ESR1	NM_000125	c.A1610G	p.Y537C	D
26	Baseline	R9	chr6:152419923	A	G	1.00	exonic	SNV	ESR1	NM_000125	c.A1610G	p.Y537C	D
26	Baseline	R1	chr6:152419923	A	G	0.29	exonic	SNV	ESR1	NM_000125	c.A1610G	p.Y537C	D
26	Baseline	R13	chr6:152419923	A	G	0.38	exonic	SNV	ESR1	NM_000125	c.A1610G	p.Y537C	D
26	Baseline	R14	chr6:152419923	A	G	0.79	exonic	SNV	ESR1	NM_000125	c.A1610G	p.Y537C	D
26	Baseline	R15	chr6:152419923	A	G	0.64	exonic	SNV	ESR1	NM_000125	c.A1610G	p.Y537C	D
26	Baseline	R19	chr6:152419923	A	G	0.64	exonic	SNV	ESR1	NM_000125	c.A1610G	p.Y537C	D
26	Baseline	R2	chr6:152419923	A	G	1.00	exonic	SNV	ESR1	NM_000125	c.A1610G	p.Y537C	D
26	Baseline	R21	chr6:152419923	A	G	0.33	exonic	SNV	ESR1	NM_000125	c.A1610G	p.Y537C	D
26	Baseline	R9	chr4:153249456	C	A	0.55	exonic	SNV	FBXW7	NM_033632	c.G1322T	p.R441L	D
26	Baseline	R1	chr10:123243219	G	T	0.19	exonic	SNV	FGFR2	NM_000141	c.C2294A	p.T765N	VUS
26	Baseline	R7	chr19:10599875	G	T	0.54	exonic	stopgain	KEAP1	NM_012289	c.C1701A	p.Y567X	D
26	Baseline	R1	chr2:48028195	A	G	0.13	exonic	SNV	MSH6	NM_000179	c.A3073G	p.R1025G	VUS
26	Baseline	R21	chr3:178938896	ATTAAC	A	0.58	exonic	stopgain	PIK3CA	NM_006218	c.2138_2142del	p.N714X	VUS
26	Baseline	R15	chr1:161298214	G	T	0.16	exonic	stopgain	SDHC	NM_003001	c.G106T	p.E36X	D
26	Baseline	R13	chr11:113935169	G	T	0.33	exonic	stopgain	ZBTB16	NM_006006	c.G1147T	p.E383X	D
26	Discontinuation	R27	chr5:112157661	G	T	0.10	exonic	stopgain	APC	NM_000038	c.G1381T	p.E461X	D
26	Discontinuation	R21	chr13:32906435	T	G	0.62	exonic	SNV	BRCA2	NM_000059	c.T820G	p.F274V	VUS
26	Discontinuation	R21	chr13:32906994	A	C	0.34	exonic	SNV	BRCA2	NM_000059	c.A1379C	p.N460T	VUS
26	Discontinuation	R9	chr13:32913216	A	G	0.12	exonic	SNV	BRCA2	NM_000059	c.A4724G	p.D1575G	VUS
26	Discontinuation	R19	chr13:32936802	G	C	0.30	exonic	SNV	BRCA2	NM_000059	c.G7948C	p.E2650Q	VUS
26	Discontinuation	R20	chr13:32936802	G	C	0.49	exonic	SNV	BRCA2	NM_000059	c.G7948C	p.E2650Q	VUS
26	Discontinuation	R21	chr13:32936802	G	C	0.91	exonic	SNV	BRCA2	NM_000059	c.G7948C	p.E2650Q	VUS
26	Discontinuation	R22	chr13:32936802	G	C	0.45	exonic	SNV	BRCA2	NM_000059	c.G7948C	p.E2650Q	VUS
26	Discontinuation	R3	chr13:32936802	G	C	0.37	exonic	SNV	BRCA2	NM_000059	c.G7948C	p.E2650Q	VUS
26	Discontinuation	R6	chr13:32936802	G	C	1.00	exonic	SNV	BRCA2	NM_000059	c.G7948C	p.E2650Q	VUS
26	Discontinuation	R12	chr13:32936802	G	C	0.41	exonic	SNV	BRCA2	NM_000059	c.G7948C	p.E2650Q	VUS
26	Discontinuation	R14	chr13:32936802	G	C	0.86	exonic	SNV	BRCA2	NM_000059	c.G7948C	p.E2650Q	VUS
26	Discontinuation	R18	chr13:32936802	G	C	0.53	exonic	SNV	BRCA2	NM_000059	c.G7948C	p.E2650Q	VUS
26	Discontinuation	R28	chr13:32936802	G	C	0.51	exonic	SNV	BRCA2	NM_000059	c.G7948C	p.E2650Q	VUS
26	Discontinuation	R30	chr13:32936802	G	C	0.62	exonic	SNV	BRCA2	NM_000059	c.G7948C	p.E2650Q	VUS
26	Discontinuation	R5	chr13:32936802	G	C	0.94	exonic	SNV	BRCA2	NM_000059	c.G7948C	p.E2650Q	VUS
26	Discontinuation	R9	chr13:32936802	G	C	1.00	exonic	SNV	BRCA2	NM_000059	c.G7948C	p.E2650Q	VUS
26	Discontinuation	R19	chr16:68842724	TG	T	1.00	exonic	frameshift deletion	CDH1	NM_004360	c.660delG	p.L220fs	D
26	Discontinuation	R20	chr16:68842724	TG	T	1.00	exonic	frameshift deletion	CDH1	NM_004360	c.660delG	p.L220fs	D
26	Discontinuation	R21	chr16:68842724	TG	T	1.00	exonic	frameshift deletion	CDH1	NM_004360	c.660delG	p.L220fs	D
26	Discontinuation	R3	chr16:68842724	TG	T	1.00	exonic	frameshift deletion	CDH1	NM_004360	c.660delG	p.L220fs	D

Patient #	Timepoint	CTC ID	Genomic location	Reference allele	Variant allele	Variant fraction	Exonic/Other	Function	Gene	RefSeq ID	Nucleotide position	Amino acid	Putative driver (D) / VUS
26	Discontinuation	R12	chr16:68842724	TG	T	1.00	exonic	frameshift deletion	CDH1	NM_004360	c.660delG	p.L220fs	D
26	Discontinuation	R13	chr16:68842724	TG	T	1.00	exonic	frameshift deletion	CDH1	NM_004360	c.660delG	p.L220fs	D
26	Discontinuation	R14	chr16:68842724	TG	T	1.00	exonic	frameshift deletion	CDH1	NM_004360	c.660delG	p.L220fs	D
26	Discontinuation	R18	chr16:68842724	TG	T	1.00	exonic	frameshift deletion	CDH1	NM_004360	c.660delG	p.L220fs	D
26	Discontinuation	R26	chr16:68842724	TG	T	1.00	exonic	frameshift deletion	CDH1	NM_004360	c.660delG	p.L220fs	D
26	Discontinuation	R27	chr16:68842724	TG	T	1.00	exonic	frameshift deletion	CDH1	NM_004360	c.660delG	p.L220fs	D
26	Discontinuation	R28	chr16:68842724	TG	T	0.99	exonic	frameshift deletion	CDH1	NM_004360	c.660delG	p.L220fs	D
26	Discontinuation	R30	chr16:68842724	TG	T	1.00	exonic	frameshift deletion	CDH1	NM_004360	c.660delG	p.L220fs	D
26	Discontinuation	R4	chr16:68842724	TG	T	1.00	exonic	frameshift deletion	CDH1	NM_004360	c.660delG	p.L220fs	D
26	Discontinuation	R7	chr16:68842724	TG	T	1.00	exonic	frameshift deletion	CDH1	NM_004360	c.660delG	p.L220fs	D
26	Discontinuation	R9	chr16:68842724	TG	T	1.00	exonic	frameshift deletion	CDH1	NM_004360	c.660delG	p.L220fs	D
26	Discontinuation	R18	chr12:58145430	C	T	0.13	exonic	SNV	CDK4	NM_000075	c.G71A	p.R24H	D
26	Discontinuation	R19	chr6:152419923	A	G	0.77	exonic	SNV	ESR1	NM_000125	c.A1610G	p.Y537C	D
26	Discontinuation	R20	chr6:152419923	A	G	0.17	exonic	SNV	ESR1	NM_000125	c.A1610G	p.Y537C	D
26	Discontinuation	R21	chr6:152419923	A	G	0.68	exonic	SNV	ESR1	NM_000125	c.A1610G	p.Y537C	D
26	Discontinuation	R3	chr6:152419923	A	G	0.13	exonic	SNV	ESR1	NM_000125	c.A1610G	p.Y537C	D
26	Discontinuation	R6	chr6:152419923	A	G	0.48	exonic	SNV	ESR1	NM_000125	c.A1610G	p.Y537C	D
26	Discontinuation	R12	chr6:152419923	A	G	1.00	exonic	SNV	ESR1	NM_000125	c.A1610G	p.Y537C	D
26	Discontinuation	R13	chr6:152419923	A	G	1.00	exonic	SNV	ESR1	NM_000125	c.A1610G	p.Y537C	D
26	Discontinuation	R14	chr6:152419923	A	G	0.66	exonic	SNV	ESR1	NM_000125	c.A1610G	p.Y537C	D
26	Discontinuation	R18	chr6:152419923	A	G	0.34	exonic	SNV	ESR1	NM_000125	c.A1610G	p.Y537C	D
26	Discontinuation	R26	chr6:152419923	A	G	0.25	exonic	SNV	ESR1	NM_000125	c.A1610G	p.Y537C	D
26	Discontinuation	R27	chr6:152419923	A	G	0.75	exonic	SNV	ESR1	NM_000125	c.A1610G	p.Y537C	D
26	Discontinuation	R28	chr6:152419923	A	G	0.50	exonic	SNV	ESR1	NM_000125	c.A1610G	p.Y537C	D
26	Discontinuation	R4	chr6:152419923	A	G	1.00	exonic	SNV	ESR1	NM_000125	c.A1610G	p.Y537C	D
26	Discontinuation	R5	chr6:152419923	A	G	0.58	exonic	SNV	ESR1	NM_000125	c.A1610G	p.Y537C	D
26	Discontinuation	R7	chr6:152419923	A	G	0.42	exonic	SNV	ESR1	NM_000125	c.A1610G	p.Y537C	D
26	Discontinuation	R9	chr6:152419923	A	G	0.35	exonic	SNV	ESR1	NM_000125	c.A1610G	p.Y537C	D
26	Discontinuation	R30	chr1:65339121	C	A	0.16	exonic	stopgain	JAK1	NM_002227	c.G415T	p.E139X	D
26	Discontinuation	R18	chr19:10610596	G	T	0.75	exonic	stopgain	KEAP1	NM_012289	c.C114A	p.C38X	D
26	Discontinuation	R16	chr3:52582143	A	G	0.26	exonic	SNV	PBRM1	NM_018313	c.T4685C	p.L1562P	VUS
26	Discontinuation	R5	chr5:67588954	G	T	0.25	exonic	SNV	PIK3R1	NM_181523	c.G1045T	p.D349Y	VUS
26	Discontinuation	R16	chr1:214531269	A	C	0.36	exonic	SNV	PTPN14	NM_005401	c.T3560G	p.I1187S	VUS
26	Discontinuation	R21	chr1:214531269	A	C	0.67	exonic	SNV	PTPN14	NM_005401	c.T3560G	p.I1187S	VUS
26	Discontinuation	R16	chr1:214542934	G	T	0.15	exonic	SNV	PTPN14	NM_005401	c.C3137A	p.T1046K	VUS
26	Discontinuation	R26	chr1:214556674	C	CTA	0.17	exonic	stopgain	PTPN14	NM_005401	c.2523_2524ins	p.E842X	D
26	Discontinuation	R12	chr3:47162426	C	A	0.14	exonic	stopgain	SETD2	NM_014159	c.G3700T	p.E1234X	D
26	Discontinuation	R21	chrX:123205129	T	G	0.10	exonic	SNV	STAG2	NM_006603	c.T2489G	p.L830W	VUS
26	Discontinuation	R22	chrX:123205129	T	G	0.12	exonic	SNV	STAG2	NM_006603	c.T2489G	p.L830W	VUS
26	Discontinuation	R21	chr9:135771774	C	A	0.49	exonic	stopgain	TSC1	NM_000368	c.G3343T	p.E1115X	D
26	Discontinuation	R21	chr9:135772901	G	A	0.98	exonic	SNV	TSC1	NM_000368	c.C2722T	p.R908W	VUS
26	Discontinuation	R22	chr9:135772901	G	A	0.18	exonic	SNV	TSC1	NM_000368	c.C2722T	p.R908W	VUS
26	Discontinuation	R9	chr9:135772901	G	A	0.12	exonic	SNV	TSC1	NM_000368	c.C2722T	p.R908W	VUS
31	Baseline	R6	chr13:32906435	T	G	1.00	exonic	SNV	BRCA2	NM_000059	c.T820G	p.F274V	VUS
31	Baseline	R7	chr6:152419926	A	G	0.45	exonic	SNV	ESR1	NM_000125	c.A1613G	p.D538G	D
31	Baseline	R4	chr17:17127344	G	T	0.11	exonic	stopgain	FLCN	NM_144997	c.C510A	p.Y170X	D
31	Baseline	R6	chr1:65301100	G	T	0.12	exonic	stopgain	JAK1	NM_002227	c.C3348A	p.C1116X	D
31	Baseline	R7	chr3:178952085	A	G	0.80	exonic	SNV	PIK3CA	NM_006218	c.A3140G	p.H1047R	D
31	Baseline	R7	chr5:236628	C	T	0.48	exonic	SNV	SDHA	NM_004168	c.C1346T	p.A449V	VUS
34	Baseline	D6	chr19:45854946	C	G	0.22	exonic	SNV	ERCC2	NM_000400	c.G2224C	p.E742Q	VUS
34	Baseline	C6	chr10:123258034	A	T	0.20	exonic	SNV	FGFR2	NM_000141	c.T1647A	p.N549K	D
34	Baseline	D6	chr1:65321333	G	A	0.13	exonic	stopgain	JAK1	NM_002227	c.C1507T	p.Q503X	D
34	Baseline	D6	chr19:10602580	C	T	0.20	exonic	SNV	KEAP1	NM_012289	c.G998A	p.G333D	D
34	Baseline	H3	chr2:48026430	C	G	0.15	exonic	stopgain	MSH6	NM_000179	c.C1308G	p.Y436X	D
34	Baseline	C6	chr3:178952085	A	G	0.22	exonic	SNV	PIK3CA	NM_006218	c.A3140G	p.H1047R*	D
34	Baseline	D6	chr3:178952085	A	G	0.33	exonic	SNV	PIK3CA	NM_006218	c.A3140G	p.H1047R*	D
34	Baseline	H3	chr3:178952085	A	G	0.75	exonic	SNV	PIK3CA	NM_006218	c.A3140G	p.H1047R*	D
34	Baseline	F3	chr3:178952085	A	G	0.48	exonic	SNV	PIK3CA	NM_006218	c.A3140G	p.H1047R*	D
34	Baseline	E6	chr3:178952085	A	G	0.36	exonic	SNV	PIK3CA	NM_006218	c.A3140G	p.H1047R*	D

Patient #	Timepoint	CTC ID	Genomic location	Reference allele	Variant allele	Variant fraction	Exonic/Other	Function	Gene	RefSeq ID	Nucleotide position	Amino acid	Putative driver (D) / VUS
34	Baseline	C6	chr17:47696689	A	G	0.10	exonic	SNV	SPOP	NM_003563	c.T259C	p.Y87H	D
34	Baseline	H3	chr9:135772722	G	A	0.12	exonic	stopgain	TSC1	NM_000368	c.C2824T	p.Q942X	D
34	Discontinuation	D6	chr5:112103068	G	T	0.13	exonic	stopgain	APC	NM_000038	c.G403T	p.E135X	D
34	Discontinuation	A9	chr11:108139278	TTGTTAA	T	0.20	exonic	framepreserving deletion	ATM	NM_000051	c.2780_2785del	p.L928_M929del	VUS
34	Discontinuation	B9	chr17:41209068	C	A	0.16	splicing	splice acceptor	BRCA1	NM_007294	c.5277 +1 G>T	exon20 +1 G>T	VUS
34	Discontinuation	E9	chr5:98235307	TTTAA	T	0.23	exonic	frameshift deletion	CHD1	NM_001270	c.959_962del	p.L319fs	D
34	Discontinuation	G3	chr5:98235307	TTTAA	T	0.12	exonic	frameshift deletion	CHD1	NM_001270	c.959_962del	p.L319fs	D
34	Discontinuation	B6	chr19:45854946	C	G	0.31	exonic	SNV	ERCC2	NM_000400	c.G2224C	p.E742Q	VUS
34	Discontinuation	D3	chr10:123258034	A	T	0.29	exonic	SNV	FGFR2	NM_000141	c.T1647A	p.N549K	D
34	Discontinuation	D6	chr10:123258034	A	T	0.25	exonic	SNV	FGFR2	NM_000141	c.T1647A	p.N549K	D
34	Discontinuation	E6	chr10:123258034	A	T	0.33	exonic	SNV	FGFR2	NM_000141	c.T1647A	p.N549K	D
34	Discontinuation	E6	chr2:209113112	C	A	0.33	exonic	SNV	IDH1	NM_005896	c.G395T	p.R132L	D
34	Discontinuation	C3	chr3:178952085	A	G	0.95	exonic	SNV	PIK3CA	NM_006218	c.A3140G	p.H1047R	D
34	Discontinuation	D3	chr3:178952085	A	G	0.72	exonic	SNV	PIK3CA	NM_006218	c.A3140G	p.H1047R*	D
34	Discontinuation	D6	chr3:178952085	A	G	0.71	exonic	SNV	PIK3CA	NM_006218	c.A3140G	p.H1047R*	D
34	Discontinuation	B3	chr3:178952085	A	G	0.13	exonic	SNV	PIK3CA	NM_006218	c.A3140G	p.H1047R*	D
34	Discontinuation	C9	chr3:178952085	A	G	0.13	exonic	SNV	PIK3CA	NM_006218	c.A3140G	p.H1047R*	D
34	Discontinuation	H3	chr3:178952085	A	G	0.44	exonic	SNV	PIK3CA	NM_006218	c.A3140G	p.H1047R*	D
34	Discontinuation	B9	chr3:178952085	A	G	0.47	exonic	SNV	PIK3CA	NM_006218	c.A3140G	p.H1047R*	D
34	Discontinuation	G3	chr3:178952085	A	G	0.54	exonic	SNV	PIK3CA	NM_006218	c.A3140G	p.H1047R*	D
34	Discontinuation	B6	chr3:178952085	A	G	0.50	exonic	SNV	PIK3CA	NM_006218	c.A3140G	p.H1047R*	D
34	Discontinuation	A9	chr3:178952085	A	G	0.54	exonic	SNV	PIK3CA	NM_006218	c.A3140G	p.H1047R	D
34	Discontinuation	A12	chr3:178952085	A	G	0.47	exonic	SNV	PIK3CA	NM_006218	c.A3140G	p.H1047R	D
34	Discontinuation	C9	chr3:49412883	T	C	0.17	exonic	SNV	RHOA	NM_001664	c.A140G	p.E47G	D
34	Discontinuation	E6	chr2:198266834	T	C	1.00	exonic	SNV	SF3B1	NM_012433	c.A2098G	p.K700E	D
34	Discontinuation	C12	chr9:135776176	G	A	0.13	exonic	stopgain	TSC1	NM_000368	c.C2551T	p.Q851X	D
34	Discontinuation	A9	chrX:70470298	A	G	0.18	exonic	SNV	ZMYM3	NM_005096	c.T1057C	p.C353R	VUS
35	Baseline	R7	chr19:45855589	G	A	1.00	exonic	SNV	ERCC2	NM_000400	c.C2068T	p.R690W	VUS
35	End of trial	R16	chr13:32906435	T	G	0.97	exonic	SNV	BRCA2	NM_000059	c.T820G	p.F274V	VUS
35	End of trial	R11	chr13:32913342	G	C	0.16	exonic	SNV	BRCA2	NM_000059	c.G4850C	p.S1617T	VUS
35	End of trial	R1	chr19:45855589	G	A	0.48	exonic	SNV	ERCC2	NM_000400	c.C2068T	p.R690W	VUS
35	End of trial	R11	chr19:45855589	G	A	1.00	exonic	SNV	ERCC2	NM_000400	c.C2068T	p.R690W	VUS
35	End of trial	R19	chr19:45855589	G	A	0.33	exonic	SNV	ERCC2	NM_000400	c.C2068T	p.R690W	VUS
35	End of trial	R9	chr19:45855589	G	A	0.93	exonic	SNV	ERCC2	NM_000400	c.C2068T	p.R690W	VUS
35	End of trial	R12	chr6:152419923	A	C	0.27	exonic	SNV	ESR1	NM_000125	c.A1610C	p.Y537S	D
35	End of trial	R19	chr6:152419923	A	C	0.45	exonic	SNV	ESR1	NM_000125	c.A1610C	p.Y537S	D
35	End of trial	R2	chr6:152419923	A	C	0.37	exonic	SNV	ESR1	NM_000125	c.A1610C	p.Y537S	D
35	End of trial	R9	chr6:152419923	A	C	0.89	exonic	SNV	ESR1	NM_000125	c.A1610C	p.Y537S	D
35	End of trial	R1	chr1:65310572	C	A	0.17	exonic	stopgain	JAK1	NM_002227	c.G2116T	p.E706X	D
35	End of trial	R9	chr1:65335027	CT	C	0.11	exonic	frameshift deletion	JAK1	NM_002227	c.614delA	p.K204fs	D
35	End of trial	R11	chr1:214531269	A	C	0.50	exonic	SNV	PTPN14	NM_005401	c.T3560G	p.I1187S	VUS
35	End of trial	R9	chr7:128849178	G	T	0.54	exonic	SNV	SMO	NM_005631	c.G1406T	p.C469F	D
35	End of trial	R2	chr9:135772010	TC	T	0.26	exonic	frameshift deletion	TSC1	NM_000368	c.3107delG	p.G1035fs	D
36	Baseline	R5	chr10:89653836	T	C	0.14	exonic	SNV	PTEN	NM_000314	c.T134C	p.V45A	D
44	Baseline	R7	chr15:45007895	G	C	0.97	exonic	SNV	B2M	NM_004048	c.G342C	p.K114N	VUS
44	Baseline	R2	chr1:214531269	A	C	0.15	exonic	SNV	PTPN14	NM_005401	c.T3560G	p.I1187S	VUS
44	Baseline	R7	chr1:214531269	A	C	0.99	exonic	SNV	PTPN14	NM_005401	c.T3560G	p.I1187S	VUS

Table shows scNGS somatic mutational calls for CTCs passing NGS quality filters. Variant calls are sorted by patient, early vs. late timepoint then gene name. Genomic location given in hg19 human genome reference version. Variant fraction, ratio of reads conatining variant over total reads for the position; SNV, single nucleotide variant; VUS, variant of unknown significance. Putative driver identification was made if an alteration is deleterious (nonsense / frameshift) in a tumor suppressor gene or a recurrent "hotspot" alteration in any gene in cbioportal.org ("curated set of non-redundant studies" cohort), or is annotated as oncogenic in OncoKB, or has literature support as being oncogenic. Mutations without support from any of those categories were designated as VUS. * denotes low coverage (range 10 - 30 reads)

Table S4. ScNGS somatic mutational calls for CTCs passing NGS quality filters. Variant calls are sorted by patient, early vs. late timepoint then gene name. Genomic location given in hg19 human genome reference version. Variant fraction, ratio of reads containing variant over total reads covering the position; SNV, single nucleotide variant; VUS, variant of unknown significance. Putative driver identification was made if an alteration is deleterious (nonsense / frameshift) in a tumor suppressor gene or a recurrent "hotspot" alteration in any gene in cbioportal.org ("curated set of non-redundant studies" cohort), or is annotated as oncogenic in OncoKB, or has literature support as being oncogenic. Mutations without support from any of those categories were designated as VUS. *denotes low coverage (range 10 - 30 reads)
